# Pyrylazo Dye: A Novel Azo Dye Structure with Photoinduced Proton Release and Highlighted Photophysical Properties in Biological Media

**DOI:** 10.1021/acsomega.4c06429

**Published:** 2024-12-27

**Authors:** Willy G. Santos, Lucas H. Pereira, Beatriz B. S. Ramin, Sabrina M. Botelho, Sinara T. B. Morais, Daniel R. Cardoso, Silvia H. Santagneli, Fabio F. Ferreira, Andrei Leitão, Sidney J. L. Ribeiro

**Affiliations:** a Federal University of ABC−UFABC, Av. dos Estados 5001, Santo André, SP 09210-170, Brazil; b Institute of Chemistry, São Paulo State University-UNESP, Araraquara, SP 14800-060, Brazil; c Chemical Institute of São Carlos, University of São Paulo, CP 780, São Carlos, SP 13560-970, Brazil

## Abstract

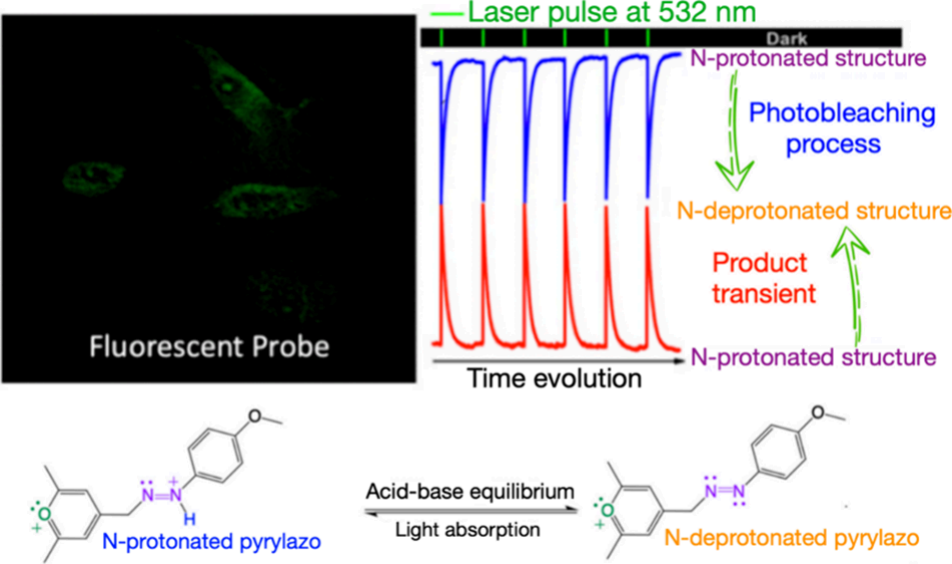

A straightforward method for synthesizing a stable, photoreactive, and fluorescent-probe azo dye molecule is presented, highlighting the influence of azo and pyrylium groups within the electronic structure of the novel dye. This compound, named the pyrylazo molecule, is synthesized through the chemical reaction between 2,4,6-trimethylpyrylium and a 4-methoxybenzenediazonium species. The methyl group at the para position of the pyrylium readily reacts with the diazonium molecule, forming a stable protonated pyrylium-azo dye (N-protonated pyrylazo). The pyrylazo structure can easily change into its N-deprotonated form upon introduction of a weak base, such as an amine, promoting significant spectral shifts in the visible absorption and fluorescence bands. Because of that and other photochemical properties, this novel dye has shown significant potential for applications in photoinduced processes and biological contexts, particularly in Coulombic interactions with micelles and animal cells. In contrast to other nonfluorescent azo dyes, the singlet excited state of pyrylazo is deactivated through a radiative process in organized media, as evidenced by its behavior during micelle media, cell membrane permeation, and fluorescence emission in the cytoplasm. Nanosecond-transient absorption spectroscopy reveals a reversible photoinduced proton release process occurring in the excited singlet state, suggesting that the excited states of pyrylazo may play roles in transport through ion channels, artificial photosynthesis, and photoinduced protein folding. These promising applications underscore the pyrylium-azo structure as a novel dye with remarkable photochemical and photophysical properties not observed in other azo dye molecules reported before.

## Introduction

Azo dyes play a crucial role in various applications due to their vibrant colors and unique properties, which are derived from the stable azo (−N=N−) functional group. This stability renders them nonphotoreactive under ultraviolet or visible-light absorption, making them reliable photostable choices for a wide range of uses in chemistry and biology, including (i) cellular imaging, (ii) pH sensing, (iii) charge, and (iv) chemical analyte detection.^1−5^ However, despite the potential for photophysical processes, reactions in the excited state are relatively rare and only reversible cis–trans isomerization is really observed in most research studies.^6−8^

Nanosecond-transient absorption (ns-TA) spectroscopy emerges as an alternative tool for exploring the dynamics in the excited states of reactive azo dyes, empowering researchers to capture the evolution of excited states, product formation, and/or equilibrium between the product and the reactant. By meticulously analyzing TA spectra and lifetimes, it is possible to investigate the formation and decay of chemical species, uncover various photophysical pathways, and evaluate how external factors like solvent polarity and specific interactions in chemical microenvironments may influence these processes. Indeed, such deep insights into the excited-state behaviors of azo dyes propel the development of innovative photoactive materials. This progress is crucial across different scientific fields, including materials science, photochemistry, and biological systems, where the benefits of light-induced molecular changes are set to drive remarkable advancements.

The presence of host–guest interaction involving azo groups or azo dyes is not uncommon, particularly in molecular recognition and supramolecular chemistry.^9−13^ Azo structures are typically classified as low or nonfluorescent due to the efficient deactivation of the excited states through isomerization processes. This nonradiative pathway has been harnessed in host–guest systems to create molecular switches, the optical properties of which have been exploited for applications in biomarker science and materials science, such as memory devices and optical switches.^14−20^

Pyrylium is another type of aromatic chemical compound with the molecular formula C_5_H_5_O^+^. It is depicted as a six-membered carbon-containing ring and one oxygen atom with a positive charge, which can be delocalized across the oxygen atom and adjacent carbon atoms, creating a stabilized aromatic system. Due to this relative stabilization, the photophysics of pyrylium compounds is typically characterized by charge transfer in the visible spectrum range and π–π* transition processes in the ultraviolet region.

Fluorescence emission within the visible spectrum range is a fundamental phenomenon associated with the excited-state deactivation of pyrylium ions. However, the photoelectron transfer (PET) process is typically observed when an adequate electron donor is in proximity to the aryl or pyrylium ring, leading to the formation of active radicals.^21,22^

Rapid conversion from pyrylium to pyridinium occurs when amine derivatives are present in the pyrylium solution. Due to this conversion and the distinct photophysical properties of the resulting pyridinium derivatives, pyrylium reactions have found applications in (i) protein and nucleic acid labeling, (ii) cellular imaging, and (iii) biosensors.^16,23−28^

**Scheme 1:**
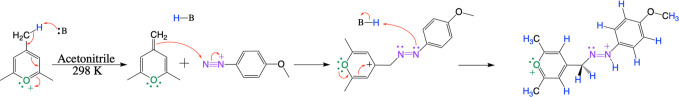
Chemical Mechanism Proposed for the Chemical Reaction between 2,4,6-Trimethylpyrylium and 4-Methoxybenzenediazonium Compounds to Form the N-Protonated Pyrylazo Molecule as an End-Product “B” is a proton receptor molecule.

**Scheme 2:**
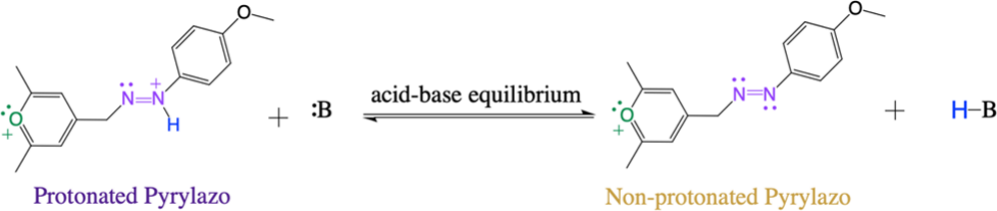
Acid–Base Equilibrium for Pyrylazo “B” is a Brønsted base.

**Scheme 3:**
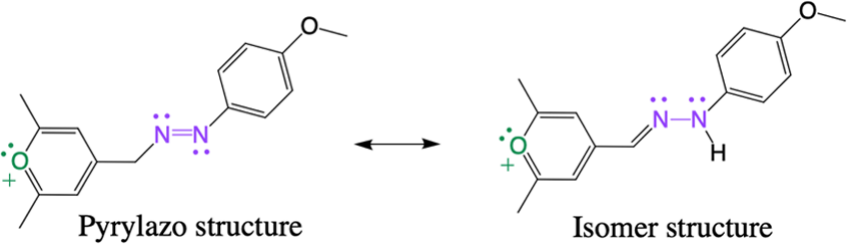
Tautomeric Equilibrium of the Pyrylazo Dye

In this study, we present notable advancements to an established synthesis route originally proposed in the 1960s^29^ as also photophysical and photochemical processes previously observed for other azo dye structures. This method enables the creation of a novel structure of an azo dye molecule by incorporating the pyrylium group close to the azo group. This integration significantly enhances the chemical versatility of the pyrylazo molecule, as observed in our photochemical and photophysical studies. Indeed, the pyrylazo structure exhibits electronic sensitivity to local polarity and Coulombic interactions with macromolecules, which affects their UV–vis spectral behavior as well as their emission properties. Additionally, a reversible photoinduced deprotonation process is also observed in solution, indicating that the dye may play roles in transport through ion channels, artificial photosynthesis, and photoinduced protein folding. The synthesis mechanism proposed is outlined in Scheme 1. Schemes 2 and 3 also show the other chemical changes proposed in this work.

This and other promising fluorescent applications underscore the pyrylium-azo structure as a novel dye with remarkable photochemical and photophysical properties not observed in other azo dye molecules reported before.

## Materials and Methods

### Chemicals

4-Methoxy benzene diazonium tetrafluoroborate (98%), sodium tetra(phenyl)borate (>99%), sodium tetra(*p*-tolyl)borate, acetonitrile (ACN), Tween 20, Triton X-100, sodium dodecyl sulfate (SDS), cetyltrimethylammonium chloride (CTAC), benzyl dimethyl hexadecyl ammonium chloride (BHAC), and other organic solvents with HPLC grade were used as purchased from Sigma-Aldrich. 2,4,6-Trimethylpyrylium tetrafluorborate (>98%) was purchased from TCI Chemicals.

### Cyclic Voltammetry

The electrochemical experiments were carried out in a three-electrode cell connected to a bipotentiostat-galvanostat μStat 400 apparatus with DropView 2.0 software from DropSens (Oviedo, Spain). The working electrode consisted of a glassy carbon disk from Metrohm (3 mm in diameter). A platinum wire and Ag/AgCl, 3 M KCl, were used as auxiliary and reference electrodes, respectively. The measurements were performed with a potential range of −1.0 to 1.0 V. All solutions were prepared in acetonitrile with tetrabutylammonium hexafluorphosphate as an electrolytic solution (0.1 M).

### UV–Vis Absorption and Emission Measurements

Absorption spectra and kinetic measurements were recorded on a Cary 5000 UV–vis–NIR spectrophotometer (Varian) at 298 K.

The fluorescence spectra were performed with an optical spectral resolution of 1 nm, using a 500–800 nm spectra window with a light source and detection slit values of 2 or 10 mm. The measurements were acquired by using a Horiba Jobin Yvon spectrofluorometer (Fluorolog-3 model FL3-122) equipped with a Hamamatsu R-928 photomultiplier tube. A cold finger accessory was used to measure at low temperatures (77 K).

A stock solution containing 1.0 × 10^–2^ M pyrylazo was prepared in acetonitrile. A few microliters (3–9 μL) of the stock solution was used to prepare other solvent solutions with a 3 mL content. Then, the UV–vis and fluorescence spectra were collected at 298 K. Equation 01 was used to determine the fluorescence quantum yield, using the safranine compound as a fluorescent standard (st) reference in acetonitrile solution.^30^
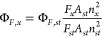
01

### FTIR-ATR Measurements

Fourier transform mid-infrared spectroscopy with attenuated total reflection (FTIR-ATR) spectra were obtained using a Hyperion 2000 from Bruker with spectra windows of 400–4000 cm^–1^, a resolution of 2 cm^–1^, and averaged 16 scans.

### Nanosecond-Transient Absorption

LFP experiments were performed in an LFP-112 ns laser flash photolysis spectrometer (Luzchem, Ottawa, Canada) using the third harmonic (532 nm) of a pulsed QSwitched Nd:YAG laser (Brilliant-B, Les Ulis, France) attenuated to 10 mJ cm^–2^ as the excitation source with 5 ns of pulse duration. The signal from the photomultiplier detection system was captured by using a Tektronix TDS 2012 digitizer (Beaverton, OR, USA). The FFP-112 ns and the digitizer were connected to a personal computer via general purpose instrumentation bus (GPIB) and serial interfaces controlling all the experimental parameters and providing suitable processing and data storage capabilities using a proprietary software package developed in a LabView environment and compiled as a standalone application (Luzchem, Ottawa, Canada). Each kinetic trace was averaged 16 times, and observed rate constants were determined by parameter fitting to the monoexponential decay function. All measurements were performed with acetonitrile (ACN) or chloroform HPLC-grade solvents purged with high-purity argon for at least 15 min before the experiments. The concentration of the protonated pyrylazo solution was adjusted to obtain an absorption value of 0.3 at 532 nm.

### NMR Measurements

NMR spectra were recorded on a Bruker Avance III HD (14.1 T) spectrometer using a nondeuterated residual signal as a reference. Nuclear magnetic resonance (^1^H NMR and ^13^C NMR) spectra and two-dimensional experiments with gradient-selected heteronuclear single quantum coherence (HSQC) were performed in acetonitrile-*d*_3_ solution (2 mg/mL).

### Mass Spectrometry Measurements

ESI-MS analyses were conducted on an ion trap mass spectrometer (Thermo Scientific, San Jose, CA, USA; model LCQ Fleet) operating in positive ion mode. Aliquots were directly infused into the ESI source at a flow rate of 10 μL min^–1^ by a micro syringe. The ESI source conditions were as follows: heated capillary temperature, 275 °C; sheath gas (N_2_) flow rate, 4 L min^–1^; spray voltage, 5.0 kV; tube lens offset voltage, 95 V. The *m*/*z* range employed in all experiments was 100–1000.

### Density Functional Theory (DFT) Calculations

All of the calculations were performed by using the Gaussian 09 Revision (G09) program package, employing density functional theory (DFT) and time-dependent (TD)-DFT methods. Calculations were run by using the Becke three-parameter hybrid functional and the Lee–Yang–Parr gradient-corrected correlation functional (B3LYP). The solvent effect was included by using the polarizable continuum model (PCM) with water as the solvent. The 6-31+G(d,p) was used for all atoms as the basis set. This same correlation function was employed for the computation of molecular structures, HOMO–LUMO orbitals, MESP surfaces, and energies of the optimized structures using Gaussian View 09 software.

### Cell Culture and Fluorescence Images

The mouse fibroblast cell line (BALB/3T3 clone A31) was cultured in DMEM supplemented with 10% fetal bovine serum (FBS) at 37 °C using a humidified incubator with 5% CO_2_ and 90% humidity. Cell passage was performed at 70% confluence.

The experiment used 5.0 × 10^4^ cells/well in tissue culture 96-well black plates with a clear flat bottom (μClear Greiner 655090). The supernatant was removed after 24 h, and the medium with the sample (pyrylazo alone or pyrylazo and 0.02% v/v Tween 20) was added to each well, following 2 h of incubation. The negative control only had a culture medium. The optimized protocol also included another step, with supernatant removal and the addition of HCS CellMask Deep Red (Thermo Fisher Scientific H32721), with 30 min of incubation. Finally, each well was washed three times with PBS and kept in 100 μL of PBS to obtain all images. Bright-field, green (λ_exc_ = 480 nm, λ_em_ = 512 nm), and red (λ_exc_ = 650 nm, λ_em_ = 655 nm) fluorescence was detected with an EvosFL epifluorescence microscope (Thermo Fisher Scientific). Experiments were performed in triplicate, and the representative image is displayed for each assay.

## Results and Discussion

### Structure Characterization and Acid–Base Equilibrium

A chemical reaction was conducted using equal molarities of 2,4,6-trimethylpyrylium and 4-methoxybenzenediazonium salts, each at a concentration of 1.0 mmol, in a 100 mL binary solvent mixture of acetonitrile and ultrapure water at a 1:1 volume ratio. The reaction was carried out in an oxygen-free environment and was stirred magnetically. Remarkably, after 30 min, the formation of the N-protonated pyrylazo molecule was confirmed by the development of a dark-purple color in the solution. The product was subsequently precipitated by evaporating the acetonitrile solvent under low-pressure and low-temperature conditions (approximately 273 K).

Upon extending the duration of the chemical reaction to 6 or 24 h, a secondary product was identified, indicating that the azo group was likely incorporated into the ortho-methyl group of the pyrylium structure. It is important to note that the investigation of this secondary product was not within the scope of this study. Further exploration of this finding may provide valuable insights into future research.

The initial product, pyrylazo, was successfully synthesized in the solid phase as microcrystals, achieving an outstanding yield of 92%. The theoretical elemental analysis confidently predicts the following values: C, 70.02%; H, 6.66%; and N, 10.89%, which align remarkably well with the experimental results of C, 65.7%; H, 6.25%; and N, 10.21%. Additionally, the calculated molar mass of pyrylazo stands at 257.14 g mol^–1^, a prediction that is confirmed by direct-injection mass spectrometry (DIMS). For further details, relevant mass data can be found in Figure S1 of the Supporting Information (SI).

**Figure 1:**
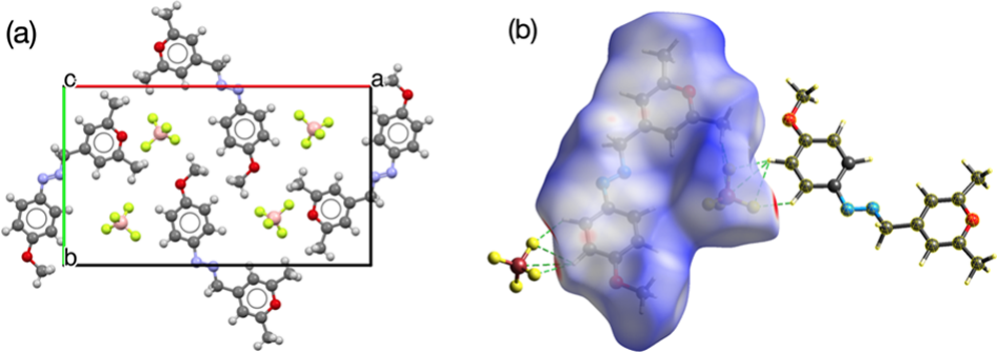
(a) Unit cell of pyrylazo (viewed along the *c*-axis) and its counterion (tetrafluoroborate). (b) Hirshfeld surface mapped over dnorm showing hydrogen bonds with neighboring tetrafluoborate anions. The surfaces are displayed as transparent to allow for visualization of the orientation and conformation of the pyrylazo structure. C–H···F hydrogen bonds are represented by green dotted lines.

The pyrylazo structure was determined through powder X-ray diffraction analysis. See Figure 1a,b for the structure.^31−35^Figure SC1 (SI) shows the blue cross symbols indicating the experimental intensities, the red line is the calculated diffractogram based on the determined crystal structure, and the gray line is the difference between the observed and calculated diffractograms. The vertical bars at the bottom indicate Bragg reflections. The result data observed in Figure SC1 was also useful in generating the Rietveld plot (Figure 1b).

The crystal structure refinement, as detailed in the Supporting Information (see the SI for Tables SC1 and SC2), indicates that the pyrylazo compound crystallizes in an orthorhombic system with the space group *Pna*21. The unit cell representation is illustrated in Figure 1a, with additional crystallographic details provided in the Supporting Information.

The FTIR spectra of the N-protonated pyrylazo compound show a moderate to low absorption in the range of 3400–3500 cm^–1^, which corresponds to the stretching mode of the N–H bond. Additionally, an aromatic carbonyl group associated with the pyrylium structure is observed at 1650 cm^–1^.

The IR signal ν(N=N^+^) at 2250 cm^–1^ from the precursor diazonium salt is completely depleted after the chemical reaction to form the pyrylazo structure (see Figure S2 in the SI). However, the exact wavenumber of the N=N band is not unequivocally identified in the FTIR spectra due to the low dipolar moment and superimposed modes of [C=C] and [C–O] occurring in the same spectral range.^36^

The ^1^H NMR and HSQC spectra of the pyrylazo compound are displayed in Figures S3 and S4 of the Supporting Information. The aromatic hydrogens exhibit peaks in the region of 7.0–8.0 ppm, with relative integration values (IV) around 2. Additionally, singlet peaks associated with the hydrogen atoms from the methoxy and methyl groups are found at 3.86 ppm (IV = 3) and 2.64 ppm (IV = 6), respectively.

Three-dimensional Hirshfeld surface plots of pyrylazo and its counterion (tetrafluoroborate anion) are shown in Figure 1b. The proximity of the fluorine atoms and two different hydrogens from the pyrylazo ring clearly reveals hydrogen bonding interactions. It is known that when a hydrogen atom is part of a hydrogen bond such as X–H···F, its isotropic ^1^H NMR shielding constant may undergo a downfield shift.

In NMR experiments conducted in the liquid phase, the hydrogen bond for all molecules of pyrylazo is not clearly observable due to the delocalization of the counterion into the solution media, which is facilitated by diffusion processes. This results in a complex multiplet signal from the hydrogen ring, encompassing both hydrogen interactions and those without. Correspondingly, the ^1^H NMR spectra of pyrylazo, along with its counterion, tetrafluoroborate, exhibit hydrogen ring signals at 7.48 and 7.04 ppm (refer to Figure S3).

The assignment of H_c_ and H_d_ protons in the pyrylazo structure was effectively explored using ^1^H NOESY and ^1^H–^13^C HMBC correlation (see Figure S4 for NOESY and Figure S5 for HMBC and HSQC spectra). In the NOESY analysis, selective irradiation at H_d_ (7.04 ppm) revealed a prominent interaction with H_e_ (from the methoxy group at 2.64 ppm) and H_c_ (7.48 ppm), indicating a significant correlation and proximity between these protons. Interestingly, selective irradiation at H_c_ (7.48 ppm) did not exhibit any observable interactions with the methoxy group, indicating a distinct separation in their environments. The HMBC experiment further corroborated these findings, showing a weaker correlation between H (H_d_) and the quaternary carbon of the methoxy group (C_q_ at δ_c_ = 159.39 ppm) compared to the stronger correlation observed with H_c_ and C_q_.

Both hydrogens from the −CH_2_ group were found at 2.18 ppm, coinciding with the position of the water signal from the solvent (acetonitrile-*d*_3_). These hydrogens within the pyrylazo structure were correlated to ^13^C through HSQC spectra, indicating the presence of hydrogens from the pyrylazo structure (see Figure S4 in the SI).

Analysis of the NOESY-1D spectra reveals that irradiation at 7.60 ppm (the hydrogen from the pyrylium ring) was sufficient to sensitize the hydrogens at 2.18 ppm (g signal) and also the hydrogen at 11.05 ppm (a signal). The same confirmation also occurred when irradiation at 2.18 ppm was performed, resulting in an increase in the intensity of the hydrogen signal at 11.05 ppm.

The hydrogen signal observed at approximately 11.05 ppm was assigned to the protonated form of the azo group N=N+(H), where the hydrogen atom is bonded to the cationic N=N+ group and is proximate to the cationic pyrylium group, thereby experiencing a stronger magnetic field. In this context, the presence of protonated or deprotonated species can be interpreted in light of an acid–base equilibrium occurring during the synthesis of pyrylazo or through an isomerization process, as illustrated in Schemes 2 and 3, respectively.

The structural integrity of both the pyrylium ring and the azo structures within the pyrylazo compound was further validated by cyclic voltammetry, which revealed two irreversible peaks at −0.47 and 0.60 V vs Ag/AgCl (Figure S6). These peaks correspond to the well-documented reduction of the pyrylium ring^37−39^ and the oxidation of the azo group,^38,40^ respectively.

The first reaction between pyrylium and diazonium ions was reported by Khromov-Borisov and Gavrilova, resulting in the formation of various isomers.^29^ Given the potential for isomerization, it was anticipated that the pyrylazo isomer could also be produced as an end-product, as illustrated in Scheme 3. However, in our experiments, when 2,4,6-trimethylpyrylium was reacted with 4-methoxybenzenediazonium in a 1:1 mol/mol ratio, only one pyrylazo structure was consistently observed as the end-product. This outcome suggests a strong preference for the formation of the pyrylazo molecule under the conditions tested, highlighting its stability and potential uniqueness in comparison to previously noted isomers.

**Figure 2:**
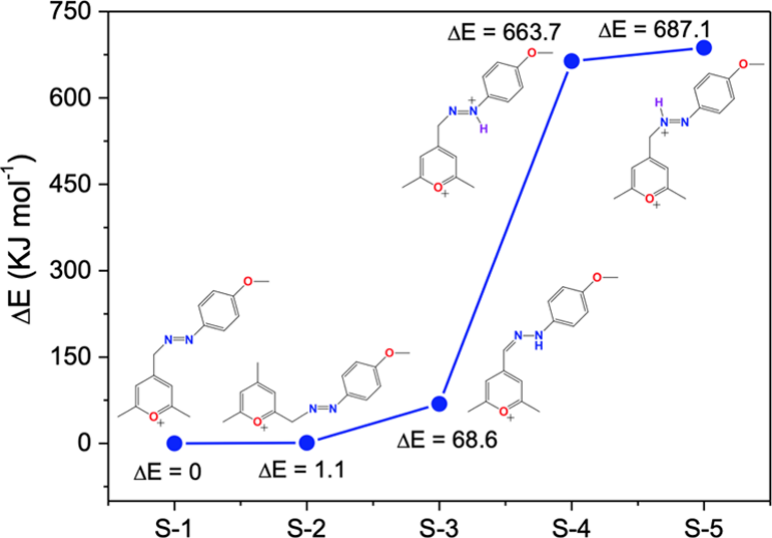
Relative Gibbs free energies of five different structures (S1–S5) were obtained through DFT calculations. The energy value of S1 was used as a reference value.

Considering the hypothesis that the pyrylazo isomer (see the S3 structure in Figure 2) or other structure configurations have the potential to be observed as end-products, the relative energies of five different possible structures (S1–S5) were evaluated through DFT calculations. As shown in Figure 2, the deprotonated pyrylazo structure (S1 structure) exhibits the lowest energy compared with the other four structures, indicating that the S1 structure (N-deprotonated pyrylazo) is the most stable. The ortho-modified structure (S2 structure) has a similar energy level to S1, suggesting that the S2 structure is a plausible end-product. However, no ortho-modified structure was detected in the characterization step (NMR, mass spectrometry, and X-ray diffraction). It is possible that the kinetics of ortho-modified pyrylazo formation may take longer than the time frame that we used in the synthesis process.

The presence of the pyrylazo isomer (S3 structure) as an end-product appears plausible, given the accessible energy observed in the theoretical results (Δ*E*_total_ = 68.6 kJ mol^–1^). However, it is important to note that a protonation process via acid–base equilibrium, as outlined in Scheme 2, cannot be dismissed and may also occur under certain conditions. This dual possibility enhances the understanding of the chemical behavior of the pyrylazo molecule in various environments.

**Figure 3:**
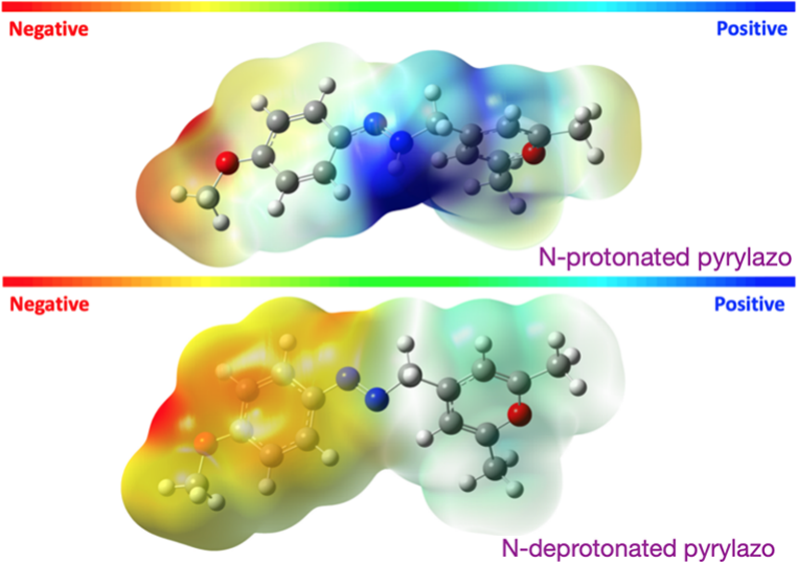
Molecular electrostatic potential map (MESP) for the N-protonated (top row) and N-deprotonated (bottom row) pyrylazo structure.

When considering the new proton, the total molecular energy experiences an increase of approximately 600 kJ mol^–1^ for the S4 and S5 structures. This change aligns with expectations from DFT calculations, as incorporating another atom (in this case, hydrogen) adds energetic bonds to the molecular structure (see Figure 2 for energy values for each structure and Figure 3 for electrostatic potential map differences between protonated and nonprotonated structures). Moreover, the increased energy level observed in the protonated pyrylazo species suggests that this protonated form is prone to proton loss when exposed to basic molecules such as amine derivatives, hydroxide anions, or HBA solvents in solution.

**Figure 4:**
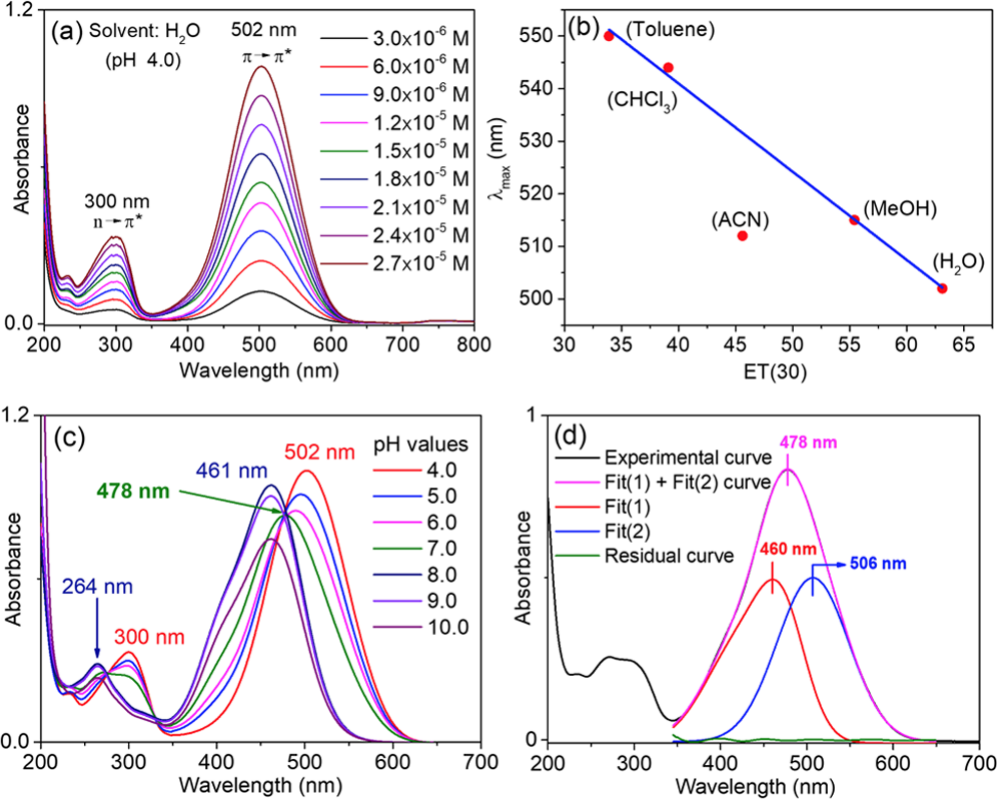
(a) Absorption spectra of protonated pyrylazo in pH 4.0 aqueous solution; (b) correlations between Reichardt’s ET(30) and the maximum absorbance values in various polar solvents; (c) absorption spectra of pyrylazo at different pH values; (d) experimental and simulated curves: Fit(1) and Fit(2) are Gaussian deconvolutions of the experimental curve; residual curve: subtraction between experimental and simulated (Fit(1) + (Fit(2))) curves.

In an aqueous solution at pH 4.0, the typical UV–vis absorption spectra characteristic of azo compounds were recorded for the protonated pyrylazo (Figure 4a). The analysis revealed two absorption bands, one at 502 nm and another at 300 nm. These were associated with a π–π* transition (where ε_(H2O)_ = 39,500 mol^–1^ L cm^–1^) and an n−π* transition (with ε_(H2O)_ = 1200 mol^–1^ L cm^–1^), respectively.

When the concentration of pyrylazo is increased, no significant changes in the absorption band shape or spectral shift were found in the UV–vis spectra, indicating the absence of any significant aggregation process.

The absorptivity constant values and the maximum absorption values of pyrylazo align with those documented for other azo dye structures in aqueous solutions or various polar solvents.^13,15,19,41,42^ However, the absorption band in the visible spectrum exhibits a blueshift as the solvent’s polarity decreases. Indeed, the plot of λ_max_ against Reichardt’s ET(30) empirical parameters^43^ demonstrates a negative solvatochromic behavior as the solvent polarity increases, indicated by a negative slope (Figure 4b). This spectral behavior implies that the π–π* transition features a significant electrostatic character contribution, with the ground state (HOMO) being more polarized than the singlet excited state (LUMO). Consequently, this leads to a larger energy gap attributed to the energetic stabilization of the ground state in polar solvents.^43,44^

In contrast to other solvents used in this work, acetonitrile (HBA solvent) does not polarize hydrogen to interact with pyrylazo. Therefore, the spectral behavior observed with ACN is expected to differ from other solvents used in this work. This distinction is crucial, as the absence of polarized hydrogen in hydrogen interactions may influence the photophysical properties and the overall response of the pyrylazo molecule in a specific solvent environment. Such differences can provide deeper insights into the solvent-specific effects on the dye’s interactions, thereby enhancing our understanding of its potential applications in distinguishing solvents.

To clarify the last hypothesis, it is essential to analyze the influence of solvent polarity on the solvatochromic shift. This requires examining various solvents with differing characteristics, including their hydrogen bond donor and acceptor abilities, solvent polarity parameters, polarizabilities, and more. However, it is important to note that several solvents react fast or slowly with pyrylazo, limiting our capacity to study its chemical behavior comprehensively.

The Coulombic nature of protonated and deprotonated pyrylazo is confirmed through DFT calculations, as illustrated in Figure 3, where positive and negative sites are represented in blue and red, respectively.

An examination of the molecular electrostatic potential map (see Figure 3) alongside the HOMO–LUMO orbitals (available in the Supporting Information) indicates that the pyrylium and azo groups play a significant role in the electronic excitation of pyrylazo. The observed π→π* transition is primarily attributed to a polarized HOMO→LUMO process, while the n→π* transition is predominantly nonpolarized.

To balance the HOMO→LUMO transition regarding the hybrid character of delocalization and polarity, a general description of the wavefunction (ψ) for the electronic absorption is provided in eq 02.
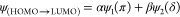
02where the coefficients α and β have varying magnitudes depending on the dipolar character of the pyrylazo molecule.

Indeed, an absorption band with hybrid character is experimentally confirmed by the hypsochromic effect, where the presence of partial positive charge into the [N=N^(+)^H] structure has strong relevance to modifying the electron density between ground and excited states. As a result, the solvent polarity modulates the optical properties of azo dyes. This solvent dependence was also observed in other azo dye structures reported in the literature.^45−47^

To understand the effect of acid–base equilibrium on the absorption spectrum of pyrylazo, the electronic absorption spectra were investigated at different pH values (pH = 4–10). Scheme 2 shows the proposed mechanism of equilibrium between the protonation and deprotonation of pyrylazo in aqueous media (Figure 4c,d).

Under acidic conditions (specifically, at pH = 4.0), the protonated form of pyrylazo is detected in the ultraviolet absorption spectra, exhibiting maximum absorbance at 502 nm (see Figure 1a). As the concentration of hydroxide ions increases (i.e., pH ≈ 8.0), the N-deprotonated form of pyrylazo is formed, leading to a corresponding decrease in the absorbance band at 502 nm associated with the N-protonated form.

**Figure 5:**
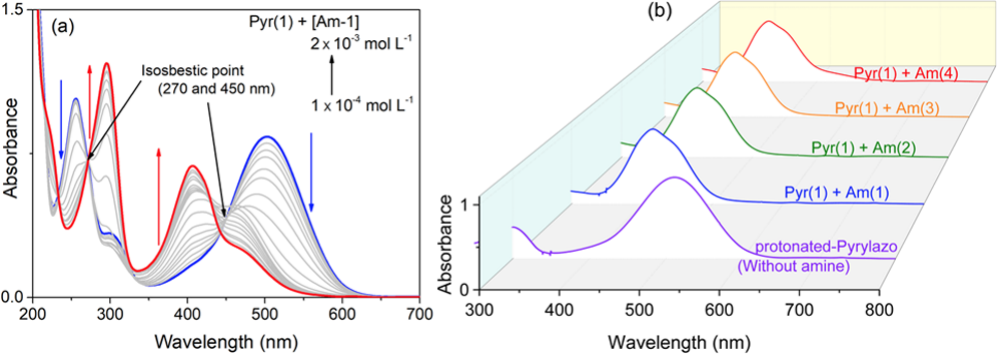
Absorption spectra of N-protonated pyrylazo (a) in the presence of varying concentrations of triethanolamine as a basic molecule or (b) in the presence of four different amines: triethanolamine (Am-1), triethylamine (Am-2), diethylamine (Am-3), and pyridine (Am-4). [Amine] = 10^–4^ mol L^–1^; [Pyr] = 3 × 10^–5^ mol L^–1^.

The absorption band for the N-deprotonated pyrylazo is observed in the range of 400–480 nm, a finding that aligns with the observations made by Martin and colleagues regarding naphthalene azo dyes.^6^ This spectral behavior under basic conditions is also evident in solutions of pyrylazo in the presence of amine molecules. See Figure 5a and Figure 5b for the absorption spectra of pyrylazo in the presence of triethanolamine and other amine compounds, respectively.

At pH 7.0, an absorption band with a maximum at 478 nm is observed (see Figure 4c, green line), which is attributed to the association of two other band components from the protonated and deprotonated species that exhibit close absorption bands This observation indicates a chemical equilibrium between the two structures (see Scheme 2). For further details, see Figure 4d, which presents the deconvoluted band spectra of each component.

**Figure 6:**
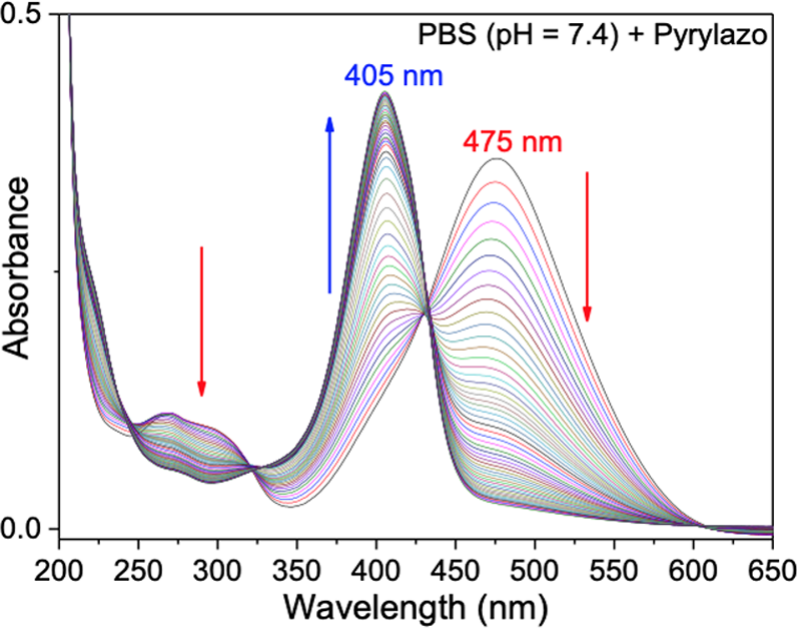
(a) Absorption spectra of N-protonated pyrylazo in the presence of a PBS buffer (pH = 7.4) recorded at different times (Δ*t* = 5 min).

When the pH is adjusted from 8 to 10, or when the pyrylazo solution is maintained under basic conditions for extended periods, the absorption band at 461 nm diminishes, while there is a corresponding increase in the band centered at 405 nm. This change is better illustrated in Figure 6, which tracks the pyrylazo absorption spectra over a duration of 6 h, with sampling intervals of 5 min, using a PBS buffer (pH 7.4) solution.

In PBS buffer conditions (see Figure 6), the absorption spectra clearly show that a prolonged and irreversible reaction with hydroxide ion is going on to form a new band centered at 405 nm. This irreversible process is also identified in amine and aqueous solutions at pH >7.4, where the absorbance value at 461 nm decreases with a concomitant increase in the absorption band around 400 nm.

The observation of a new band around 400 nm has been attributed to the ring-opening process that has been documented in previous studies concerning the reaction between amine compounds (such as hydroxide anions or other nucleophilic species) and the pyrylium structure.^27,48−50^ This observation supports the understanding of the interactions and transformations involving pyrylium, illustrating how these reactions may influence the photophysical properties of the compounds in question.

### Fluorescence in Organized Media (Micelles)

**Figure 7:**
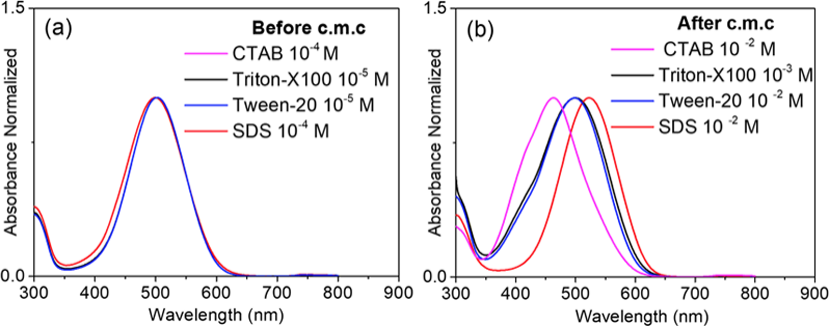
UV–vis spectra of pyrylazo (a) before and (b) after critical micellar concentration.

**Figure 8:**
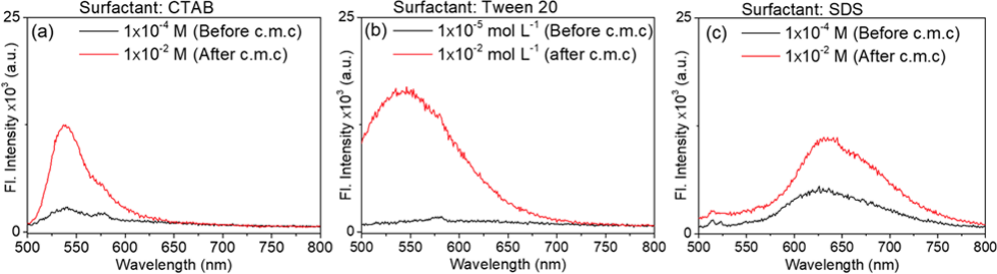
Fluorescence spectra of pyrylazo before and after cmc; surfactant system: (a) CTAB, (b) Tween 20, and (c) SDS. λ_exc_ = 480 nm.

To elucidate the photophysical properties of pyrylazo in biological media, UV–vis and fluorescence measurements were conducted within micellar systems (Figures 7 and 8). Understanding the interplay of polar and nonpolar interactions is crucial for comprehending the dynamics of colloidal systems. It is anticipated that the introduction of a surfactant to the aqueous pyrylazo solution (pH = 7.0) may significantly alter both the absorption and fluorescence spectra. As shown in Figure 7a, prior to reaching the critical micellar concentration (cmc), a low concentration of either an ionic or nonionic surfactant (1 × 10^–5^ mol L^–1^) does not produce a significant change in the absorption profile. Conversely, when the surfactant concentration exceeds the cmc., the UV–vis measurements indicate a noteworthy shift in the maximum absorption band, which is influenced by the type and charge of the surfactant present.

Wu and co-workers^51^ have observed the polarity chromism and thermochromism of micelles based on azo dye structures. However, our work introduces a new class of azo-pyrylium dye that shows a distinct interaction with micelles, indicating the presence of charge, which is not observed by Wu and co-workers.

As illustrated in Figure 7b, the maximum absorbance value exhibits a redshift in the presence of an anionic surfactant (SDS), a blueshift with a cationic surfactant (CTAC), and no shift for the two nonionic surfactants (Tween 20 and Triton X-100). These observations suggest that the changes in the electronic spectrum of pyrylazo are attributed to the electronic association of the pyrylazo with the Coulombic nature of the micellar media.

In addition to the changes observed in the UV–vis absorption spectra, there was a notable increase in fluorescence intensity after reaching the critical micelle concentration (cmc), as illustrated in Figure 8. This fluorescence increase from zero to Φ_F_ ∼0.008 (for the Tween 20 system) suggests that the pyrylazo is situated within a constrained environment, attributed to its solubilization in a relatively polar microenvironment and enhanced by strong interaction forces, such as Coulombic and/or charge-polar interactions. In pure water, fluorescence emission in the visible spectrum is not significantly observed in the absence of a surfactant.

The Coulombic interaction between pyrylazo dyes and the surface of micelles significantly hinders their relaxation processes, particularly those that are nonradiative in nature. This interaction restricts the movement along the typical axes of rotational and inversion isomerization of pyrylazo, effectively immobilizing the dyes in a specific isomer configuration. As a result of this confinement, the excited states of the dyes are unable to freely transition to other configurations. Instead, the deactivation process of pyrylazo is a radiative process (fluorescence process). Indeed, this dynamic illustrates how the microscopic interactions between the dye molecules and their environment can influence their photophysical behavior.

At low ranges of the SDS content, before cmc, the presence of emission is evidenced in the fluorescence spectra, suggesting that opposite charges between protonated pyrylazo (cationic) and SDS (anionic) may interact strongly, which may lead to the formation of a fluorescent ion-pair complex. The effect of Coulombic complexation on the photophysical and photochemical properties of pyrylazo will be better discussed at the end of the section “Photoinduced Proton Release Using Visible Light”.

In order to decrease nonradiative pathways, such as those observed in the isomerization process or other vibronic coupling steps, fluorescence measurement was performed at 77 K in the absence of surfactant molecules. As shown in Figure S8, the nonradiative pathway is inefficient at 77 K, and the fluorescence intensity is substantially increased. These results indicate that fluorescence can be observed in the pyrylazo molecule when the nonradiative pathway is restricted, as also observed in micellar media, where the chemical interaction between micelles and pyrylazo has substantial restriction on the free movement of the pyrylazo molecule.

The photophysical behaviors observed for pyrylazo in organized media (micelles) were not properly observed in other azo molecules in the literature, where the wavelength of maximum absorption was shifted depending on the Coulombic character of the organized media. In this sense, this work significantly improves the relevance of the pyrylazo molecule as a new class of azo dyes with host–guest interactions.

### Pyrylazo as a Fluorescent Probe for Cell Culture

Live-cell imaging is crucial for an improved understanding of dynamic biological processes.^52^ This technique requires probes that interact with the cell membrane, organelle, or endogenous molecules without altering their physiological and structural integrity. In this sense, the pyrylazo structure was designed to target negative biological sites and/or produce measurable signals directly correlated to biological function or activity, with various applications when functionalized with the desirable chemical. The first tests were made here using the mouse fibroblast cell lineage known as BALB/3T3 clone A31, widely used in cytotoxicity assays.^53^

The initial experiments were performed with compound concentrations during 2 h of incubation to optimize the labeling conditions without triggering cell cytotoxicity. Higher concentrations of pyrylazo (250 μM and above) led to high fluorescence but also to morphological alterations of the cell shape (Table S1). Concentrations above 3500 μM killed the cells, and no fluorescence was observed (not shown).

To avoid the cytotoxicity of pyrylazo in the cell, micelle solutions were considered to reduce what seems to be the oxidative stress effect and, second, to mitigate toxicity. However, the tests made with coadministration of the cell permeabilization surfactant Tween 20 at 0.02% did not improve the results (Table S2).^54^ Hence, the surfactant did not affect the pyrylazo penetration in the cell and consequently its cytotoxicity.

**Figure 9:**
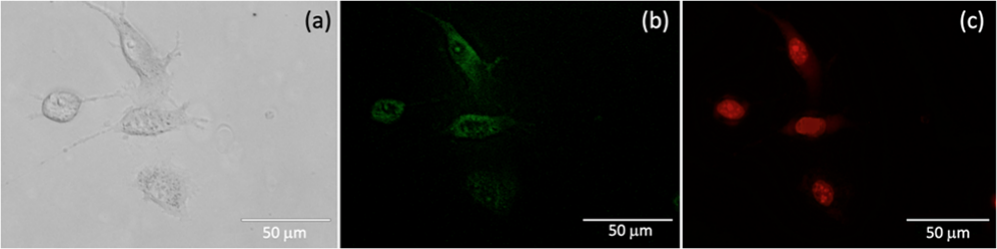
Bright-field (a) and epifluorescence (b) microscopy images of 3T3 clone A31 fibroblast cells (5.0 × 10^4^ cells/well) treated with 100 μM pyrylazo as a fluorescent probe (green). HCS CellMask Deep Red (c) was used to label the nucleus (dark red) and cytoplasm (light red) of the cells.

Next, lower concentrations of the pyrylazo were incubated with the cell, and it was observed that the fluorescence intensity was concentration-dependent (Table S3). The best result is shown in Figure 9, in which the pyrylazo fluorescence was observed throughout the cell. In this sense, the integrity and morphology of the cells may be analyzed in future works. Since the compound appears to have bonded in the cell membrane, it becomes possible to compare the morphology of defective cells with that of healthy ones, in addition to exploring numerous other applications, such as specific protein markers (hence the interest in understanding the binding targets of the molecule on the cell surface).

Although the pyrylazo compound shows promise, it demonstrated significant toxicity to the cell line in highly concentrated media. To address this, we considered the use of micelles to reduce what seems to be the oxidative stress effect and, second, to mitigate toxicity.

The efficacy of fluorescence imaging of live cells or photochemical reactions can be substantially improved in targeting by exploiting pH dependence with light or polarity of biological sites. From this perspective, photoinduced proton release processes in different polar media were investigated by ns-transient absorption.

### Photoinduced Proton Release Using Visible Light

**Figure 10:**
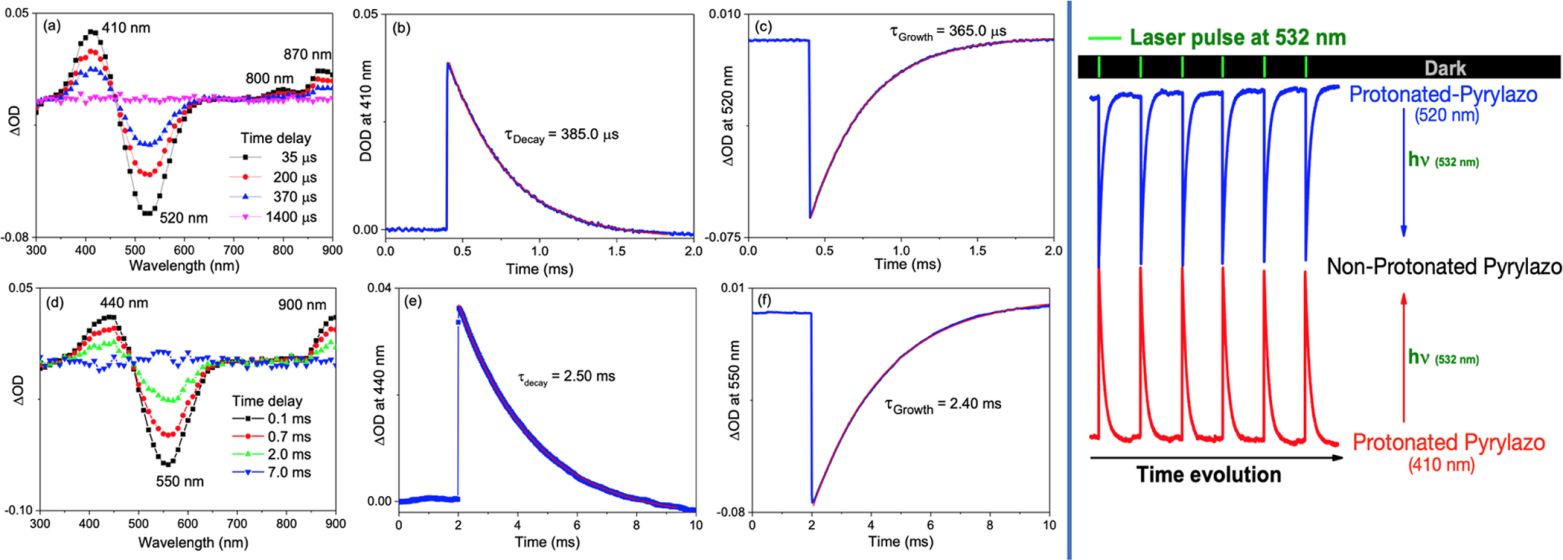
Nanosecond-transient absorption spectrum and kinetic curves of protonated pyrylazo in (a–c) acetonitrile and (d–f) chloroform solvent. λ_exc_ = 532 nm; *P* = 10 mJ cm^–2^. The right side shows a sequence of kinetic traces for the absorption transients at 520 and 410 nm.

Figure 10a shows nanosecond-transient absorption (ns-TA) spectra of protonated pyrylazo (5 × 10^–5^ mol L^–1^) in an ACN solvent by using laser excitation at 532 nm (10 mJ, 5 ns pulse). The transient spectra show two species with maximum absorption at 520 nm (negative Δ*A* signal) and 405 nm (positive ΔOD signal).

The rise time of the absorption component at 520 nm has a monoexponential growth with a lifetime of around 365 μs, which is very close to the monoexponential decay obtained for the absorption component at 410 nm with a time decay around 385 μs (see Figure 10b,c).

The similarities between both lifetimes show an inverse dependence between them, wherein the excitation of the protonated pyrylazo at 532 nm promotes photoinduced deprotonation of the N-protonated pyrylazo molecule, forming the N-deprotonated pyrylazo structure with a maximum absorption at 410 nm. A similar approach to induce pH changes after laser excitation was observed for phenolic compounds and other compounds, facilitating proton generation through the ionization of their excited state.^55,56^

When the high-polarity solvent is replaced with a low-polarity solvent such as chloroform (Figure 10d), all transient bands are redshifted, as observed in our steady-state absorption measurements. The main transient absorption in chloroform for the N-deprotonated and N-protonated pyrylazo is observed at 440 and 550 nm, respectively.

Concomitantly with the lifetime decay at 410 nm, similar lifetime decay was also observed for the absorption bands at 800 and 870 nm, indicating that these signals are due to the same nonprotonated pyrylazo molecule. In agreement with the transient absorption results in the ACN solvent, the kinetic growth and decay for these two species in chloroform are also observed with similar lifetime values (τ = 2.40–2.50 ms); see Figure 10e,f for the kinetic curves.

The shortest decay time observed for the transient at 440 nm in the acetonitrile (high-polarity) solvent, when compared to the chloroform solvent (low-polarity), is due to the solvent cage effect of a polar solvent, where the proton is held close to the N-deprotonated pyrylazo and prevented from quickly escaping by a cage of solvent molecules.

For biological purposes, the concentration of pyrylazo in the cell medium may be prepared from 0 to 1 × 10^–3^ mol L^–1^. When a laser beam at 532 nm is used as the irradiation source for 5 min in the acetonitrile solvent, around 70% of the pyrylazo molecules go to the excited state, promoting a fast deprotonation process. In this sense, for example, a 1 × 10^–4^ mol L^–1^ pyrylazo solution may generate 7 × 10^–5^ mol L^–1^ H^+^ ion in the solution (pH ∼4.2). This concentration was estimated from the maximum absorbance values of deprotonated and protonated absorption bands from laser flash photolysis measurements using a 10 mJ/cm^2^ power intensity.

When a low-intensity source, such as a xenon lamp set at 532 nm, was used in the experiments, no changes were observed in the transient spectra during 30 min of photolysis, indicating that the pH level is not significantly affected by the incidence of a few number of photons.

## Conclusions

In conclusion, we outlined a synthetic route for producing a novel photoactive pyrylium-azo dye through the chemical reaction between the trimethylpyrylium cation and diazonium salt. The result of this synthesis is a cationic pyrylium-azo dye compound referred to as a pyrylazo molecule. During the synthesis, the protonated form of pyrylazo is generated, which exhibits maximum visible absorption around 560 nm in a low-polarity solvent, such as chloroform. In a more polar solvent such as acetonitrile, a hypsochromic effect is observed for the pyrylazo solution, indicating that the polarity of the chemical microenvironments influences the optical properties of pyrylazo.

The N-protonated pyrylazo displays a visible absorption band (∼500 nm) in acidic aqueous solutions (pH < 7.0). As the pH increases, the N-deprotonated pyrylazo molecule forms, revealing a new absorption band near 450 nm, indicating its sensibility to amine compounds.

The microenvironments of ionic micelles were investigated using UV–vis absorption and fluorescence emission techniques. Once the critical micelle concentration (cmc) is reached, cationic, anionic, and neutral micelles are differentiated by the wavelength of maximum absorption of the protonated pyrylazo molecule. In sodium dodecyl sulfate (SDS) micelles, a bathochromic effect is noted, while in cetyltrimethylammonium bromide (CTAB) micelles, a hypsochromic effect is observed compared to neutral micelles. Notably, in cellular media, pyrylazo interacts significantly with various components of mouse fibroblast cells, including both membranes and organelles, suggesting its potential application as a fluorescent cellular probe.

The protonated pyrylazo species can be detected around 520 nm in the acetonitrile solvent through ns-transient absorption. Following a laser pulse at 532 nm, the N-protonated pyrylazo species transitions to N-deprotonated pyrylazo, revealing a new absorption band at approximately 410 nm.

The reversible photoinduced proton release process observed for pyrylazo in solution shows that the excited states of pyrylazo may play roles in transport through ion channels, artificial photosynthesis, and photoinduced protein folding, which are not observed in other azo dye molecules reported before.

## Supporting Information Available

Experimental details, additional NMR spectra, DFT results, X-ray diffraction, and other characterization data (PDF)
