# Supplementary material for: Pyrylazo Dye: A Novel Azo Dye Structure with Photoinduced Proton Release and Highlighted Photophysical Properties in Biological Media
Source: ACS Omega. 2024 Dec 27;10(3):2517–27. doi: 10.1021/acsomega.4c06429 (PMC11780562; doi:10.1021/acsomega.4c06429)
Supplement: Supplementary file 1 — ao4c06429_si_001.pdf [file ao4c06429_si_001.pdf]

Supplementary Information

Pyrylazo Dye: A Novel Azo Dye structure with Photoinduced Proton Release and Highlighted Photophysical Properties in biological Media

Willy G. Santos,[a,*] Lucas H. Pereira,[b] Beatriz B. S. Ramin,[b] Sabrina M. Botelho,[c] Sinara T. B. Morais,[c] Daniel R. Cardoso,[c] Silvia H. Santagneli,[b] Fabio F. Ferreira,[a] Andrei Leitão[c] and Sidney J. L. Ribeiro[b]

a. Federal University of ABC – UFABC, Av. dos Estados 5001 – Santo André – SP, 09210-170, Brazil

b. Institute of Chemistry, São Paulo State University - UNESP, Araraquara, SP 14800-060, Brazil

c. Chemical Institute of São Carlos, University of São Paulo, CP 780, São Carlos, SP 13560-970, Brazil

Summary

Experimental section……..…………………………………………………….…………..……S2

Mass spectrometry…………………………….…………………...……….……..……..S5

FTIR-ATR……….…………………………….……………………………..….………S6

NMR…….…………….………………………..……………………………....….…S7

Cyclic Voltammetry…………….…………………..…………………………….….….…S8

Uv-vis…………...………………………………………………………………….………S9

Fluorescence………………………………..……………………………….….……….…….S10

DFT Calculation Results …………………..…………………………..……...……..……...S11

Cell assays …………………..……………………….……...…….…………………………S14

Crystallographic data results…………………..…………………………..……......…………S17

Experimental section

Chemicals. 4-methoxy benzene diazonium tetrafluoroborate (98%), sodium tetra(phenyl)borate (> 99%), sodium tetra(p-toluyl)borate, acetonitrile (ACN), Tween 20, Triton X-100, Sodium Dodecyl Sulfate (SDS), Cetyltrimethylammonium chloride (CTAC), Benzyl dimethyl hexadecyl ammonium chloride (BHAC) and others organic solvents with HPLC grade were used as purchased from Sigma-Aldrich. 2,4,6-trimethylpyrylium tetrafluorborate (>98%) was purchased from TCI Chemicals.

Cyclic Voltammetry. The electrochemical experiments were carried out in a three-electrode cell connected to a Bipotentiostat-Galvanostat μStat 400 with DropView 2.0 software from DropSens (Oviedo, Spain). The working electrode consisted of a glassy carbon disk from Metrohm (3 mm in diameter). A platinum wire and Ag/AgCl, 3M KCl, was used as auxiliary and reference electrode, respectively. The measurements were performed with a potential range of -1.0 to 1.0 V. All solution was prepared in acetonitrile with tetrabutylammonium hexafluorphosphate as an electrolytic solution (0,1 M).

UV−Vis Absorption and Emission Measurements. Absorption spectra and kinetics measurements were recorded on a Cary 5000 UV-Vis-NIR spectrophotometer (Varian) at 298 K. The fluorescence spectra were performed with an optical spectral resolution of 1 nm, using a 500-800 nm spectra window with a light source and detection slit values of 2 mm. The measurements were acquired using a Horiba Jobin Yvon spectrofluorometer (Fluorolog-3 model FL3-122) equipped with a Hamamatsu R-928 photomultiplier tube. A cold-finger accessory was used to measure at low temperatures (77 K).

A stock solution containing 1.0×10^{-2} M of Pyrylazo was prepared in acetonitrile. A few microliters (3 – 9 μL) of the stock solution were used to prepare other solvent solutions with 3mL of content. Then, the UV–vis, and fluorescence spectra were collected at 298K.

The equation below was used to determine the fluorescence quantum yield, using safranine compound as fluorescent standard (st) reference in acetonitrile solution (see fluorescence properties in the article using DOI: 10.1016/j.jphotochem.2005.01.010).

$$\phi_{f,x} = \phi_{f,st} \frac{F_x}{F_{st}} \frac{A_{st}}{A_x} \frac{n_x^{\ 2}}{n_{st}^{\ 2}}$$

The equation used for fluorescence quantum yield

FTIR-ATR measurements. Fourier transform mid-infrared spectroscopy with attenuated total reflection (FT-IR/ATR) spectra were obtained using a Hyperion 2000 from Bruker with spectra windows of 400-4000 cm^{-1}, resolution of 2 $cm^{-1,}$ and averaged 16 scans.

ns-Transient absorption (Laser Flash Photolysis). LFP experiments were performed in an LFP-112 ns laser flash photolysis spectrometer (Luzchem Ottawa, Canada) using the third harmonic (532 nm) of a pulsed QSwitched Nd:YAG laser (Brilliant-B, LesUlis, France) attenuated to 10 mJ cm^{-2} as the excitation source with 5 ns of pulse duration. The signal from the photomultiplier detection system was captured using a Tektronix TDS 2012 digitizer (Beaverton, OR, USA). The FFP-112 ns and the digitizer were connected to a personal computer via General Purpose Instrumentation Bus (GPIB) and serial interfaces controlling all the experimental parameters and providing suitable processing and data storage capabilities using a proprietary software package developed in a LabView environment and compiled as a stand-alone application (Luzchem, Ottawa, Canada). Each kinetic trace was averaged 16 times, and observed rate constants were determined by parameter fitting to the mono-exponential decay function. All measurements were performed with acetonitrile (ACN) or chloroform HPLC grade solvents purged with high-purity argon for at least 15 min before the experiments. The concentration of protonated-Pyrylazo solution was adjusted to obtain an absorption value of 0.3 at 532 nm.

NMR measurements. NMR spectra were recorded on Bruker Avance III HD (14.1T) spectrometer using a nondeuterated residual signal as a reference. Nuclear magnetic resonance (^{1}H NMR and ^{13}C NMR) spectra and two-dimensional experiments with gradient-selected heteronuclear single quantum coherence (HSQC) were performed in acetonitrile-D3 solution (2mg/mL).

Mass spectrometry measurements. ESI-MS analyses were conducted on an ion trap mass spectrometer (Thermo Scientific, San Jose, CA, USA; model: LCQ Fleet) operating in the positive ion mode. Aliquots were directly infused into the ESI source at a flow rate of 10 μL min^{-1} by a micro syringe. The ESI source conditions were as follows: heated capillary temperature 275 ºC; sheath gas (N_2) flow rate 4 L min^{-1}; spray voltage 5.0 kV; tube lens offset voltage 95 V. The m/z range employed in all experiments was 100-1000.

Density Function Theory (DFT) Calculations. All the calculations were performed by using the Gaussian 09 Revision (G09) program package, employing density functional theory (DFT) and time-dependent (TD)-DFT methods. Calculations were run using the Becke three-parameter hybrid functional and the Lee-Yang-Parr gradient-corrected correlation functional (B3LYP). The solvent effect was included using the polarizable continuum model (PCM) with water as the solvent. The 6-31+G(d,p) was used for all atoms as the basis set. This same correlation function was employed for the computation of molecular structures, HOMO–LUMO orbitals, MESP surfaces, and energies of the optimized structures by using the Gaussian View 09 software.

Cell culture and fluorescence images. The mouse fibroblast cell line (Balb/3T3 clone A31) was cultured in DMEM supplemented with 10% fetal bovine serum (FBS) at 37 ºC using humidified incubator with 5% CO_2 and 90% humidity. Cell passage was performed at 70% confluence.

The experiment used $5.0x10^4$ cells/well in tissue culture 96-well black plate with a clear flat bottom (µClear® Greiner 655090). The supernatant was removed after 24 h, and the medium with the sample (Pyrylazo alone, or Pyrylazo and 0.02% v/v Tween-20) was added to each well, following 2 h incubation. The negative control only had culture medium. The optimized protocol also included another step, with supernatant removal and the addition of HCS CellMask™ Deep Red (ThermoFisher Scientific H32721), with 30 min incubation. Finally, each well was washed three times with PBS and kept in 100 µL PBS to obtain all images. Bright-field, green (λ_{exc} = 480 nm, λ_{em} = 512 nm), and red (λ_{exc} = 650 nm, λ_{em} = 655 nm) fluorescence were detected with an EvosFL epifluorescence microscope (Thermo Fisher Scientific). Experiments were performed in triplicate, and the representative image is displayed for each assay.

Mass spectrometry

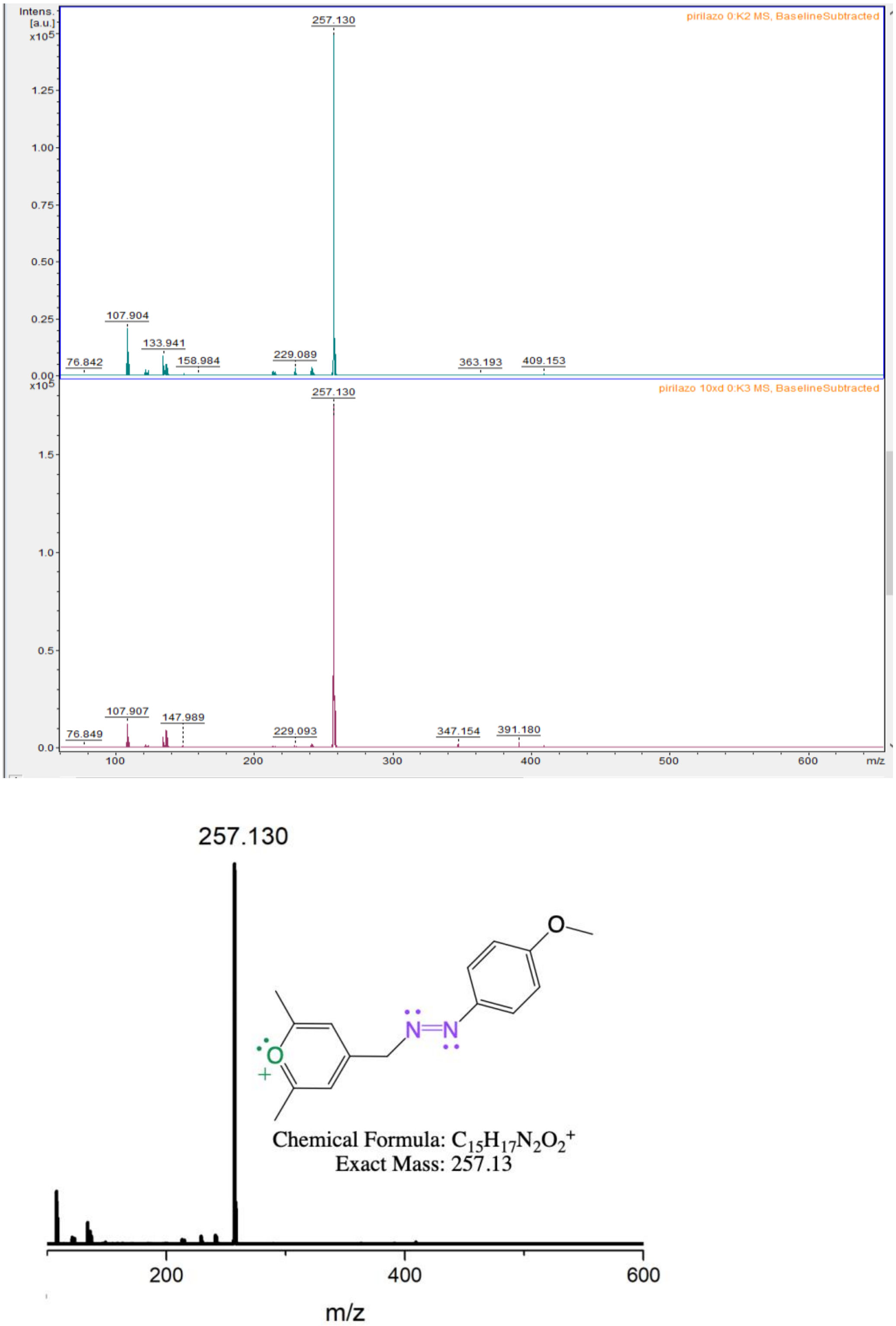

Figure S1. Mass spectrum of the Pyrylazo molecule.

FTIR/ATR

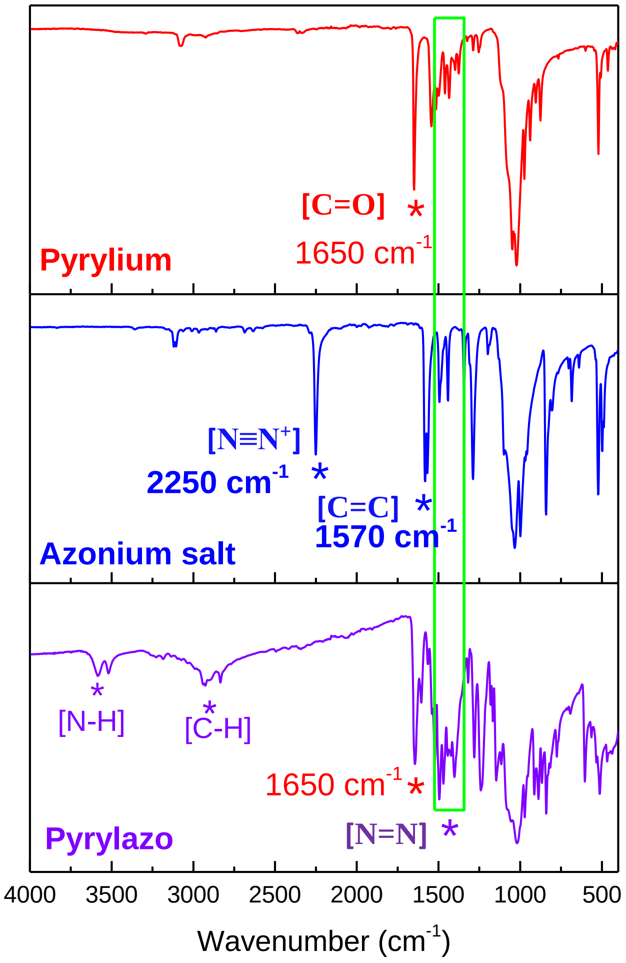

Figure S2. Fourier transform infrared spectroscopy (FTIR) spectra of Pyrylazo and its precursors (pyrylium and diazonium salts).

NMR resuts

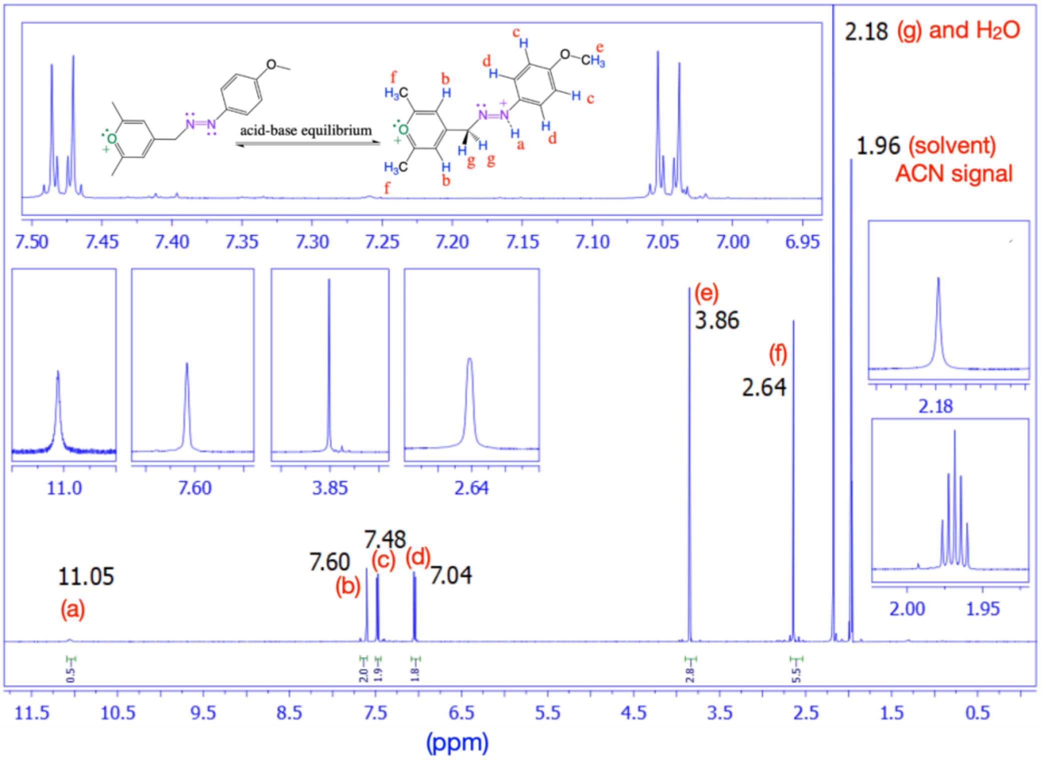

FigureS3. ^{1}H-NMR spectra of Pyrylazo in acetonitrile-d^{3} (2mg/mL).

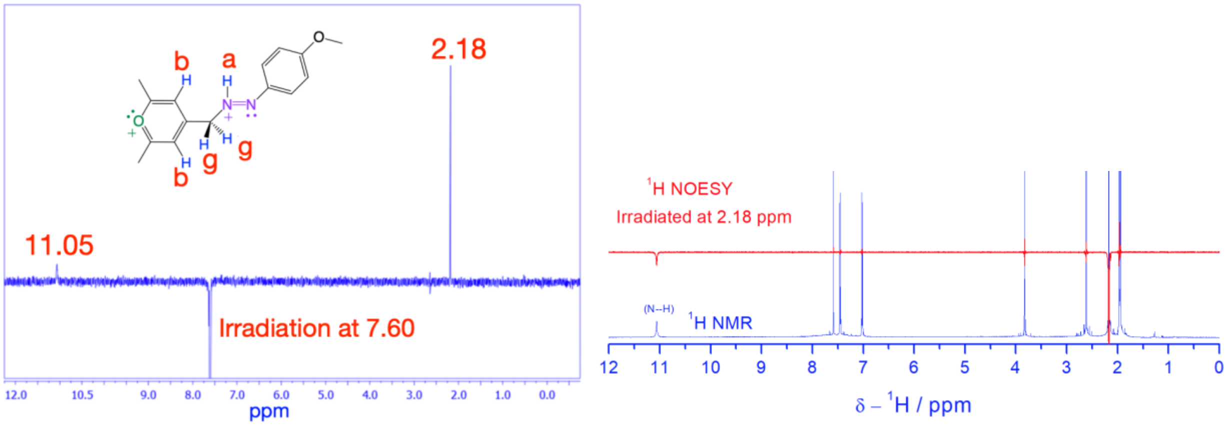

Figure S4. (left side) NOESY-1D spectra of Pyrylazo irradiated at 7.61 ppm. (right side) NOESY-1D spectra of Pyrylazo irradiated at 2.18 ppm. Acetronitrile-d3 was used as a solvent.

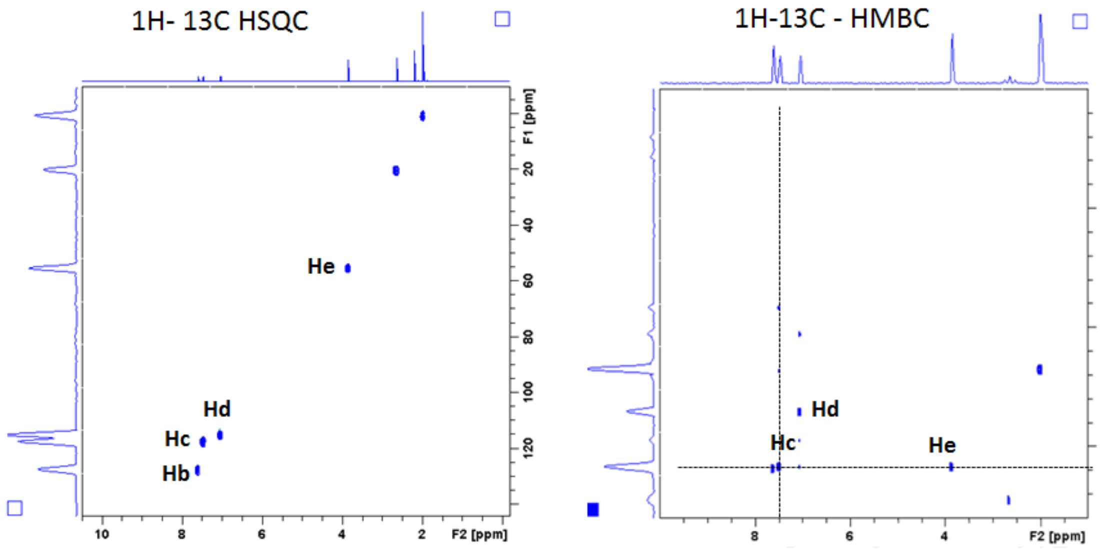

Figure S5. HSQC and HMBC spectra of Pyrylazo. Acetronitrile-d3 was used as a solvent.

Cyclic voltammetry

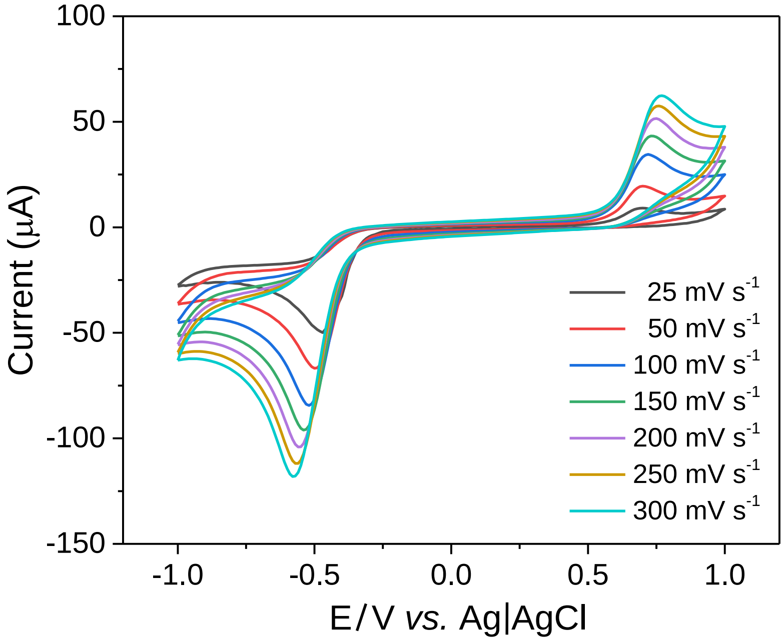

Figure S6. The effect of cyclic voltammetry scan rate on the Pyrylazo (10×10^{-3} mol L^{-1}) in ACN solvent, using tetrabutylammonium hexafluorophosphate ($NBu_4 PF_6$) (0.1 mol L^{-1}) as supporting electrolyte, platinum wire as working and counter electrode, and saturated Ag/AgCl as reference electrode. The potential was swept from -1.0 to 1.0 V with different scan rates.

UV-vis Absorption Results

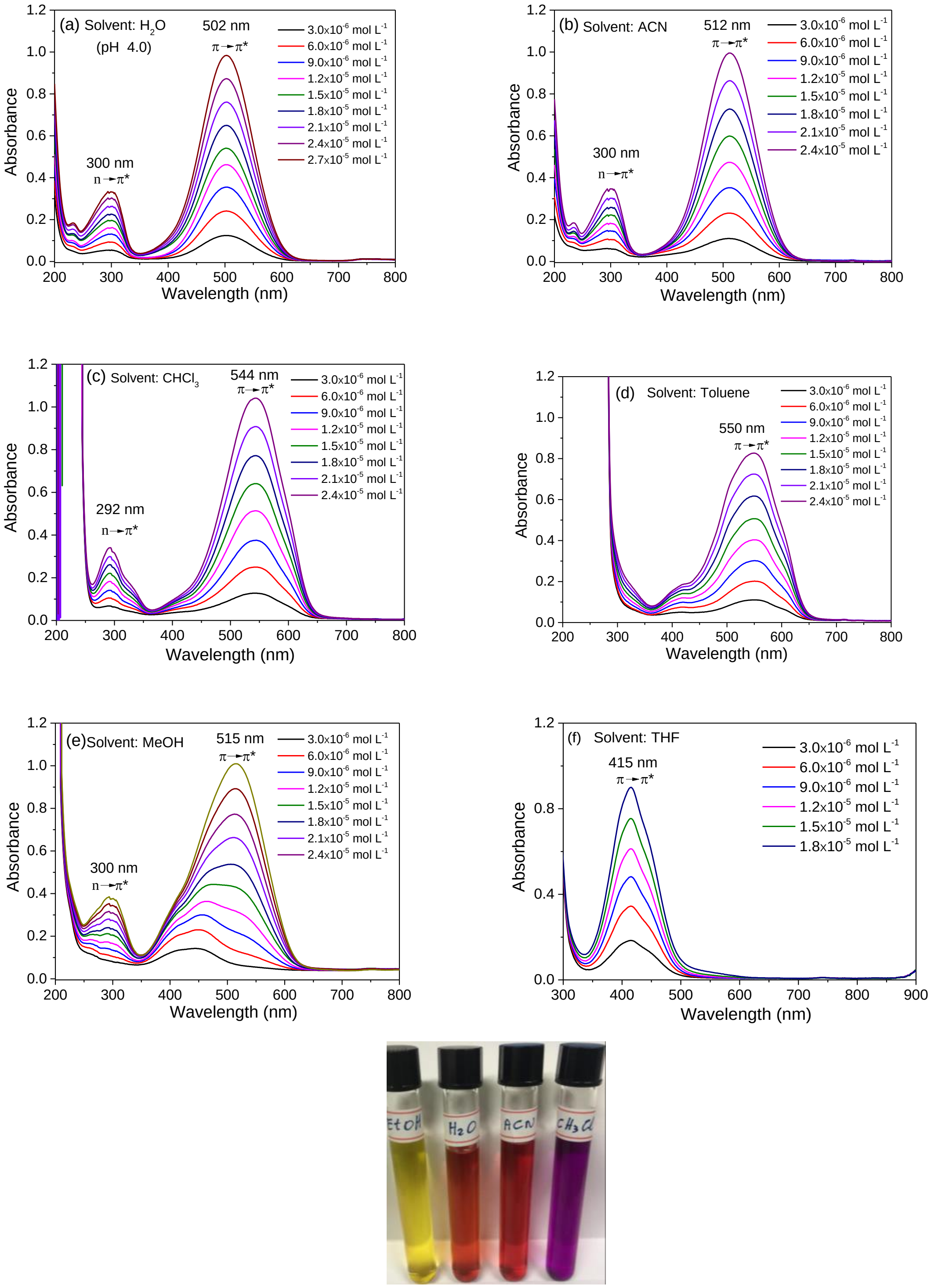

Figure S7. UV-vis absorption spectra of Pyrylazo using (a) H_2O - pH = 4.0, (b) acetonitrile - ACN, (c) chloroform, (d) toluene, (e) methanol and (f) chroloform as organic solvents. The last picture shows the solution colors using different solvents: EtOH, H_2O, ACN, and $CHCl_3$.

Fluorescence Results

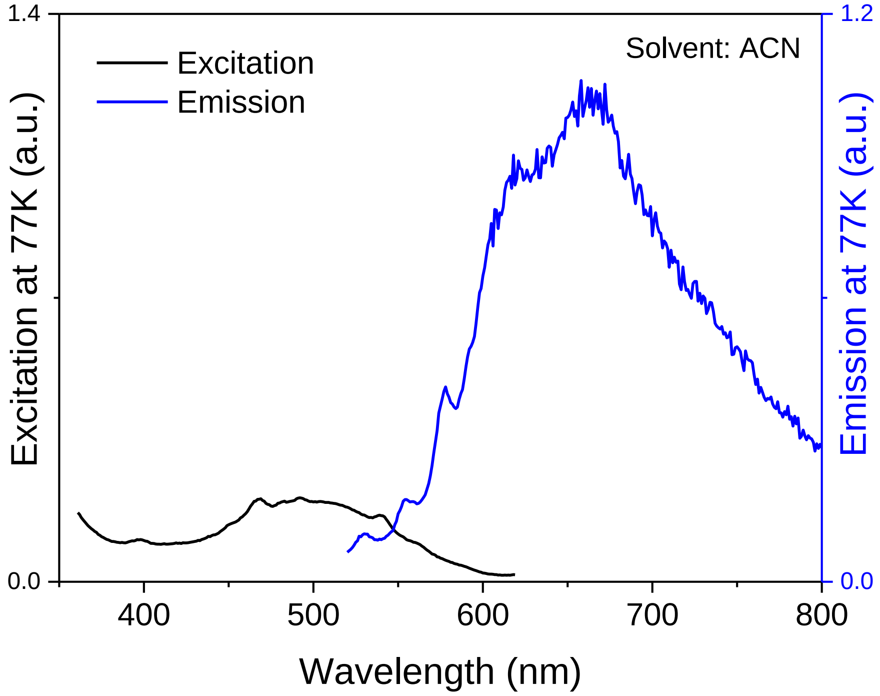

Figure S8. Front-face excitation and emission spectra of protonated Pyrylazo at 77 K, using ACN as solvent. λ_{exc} = 480 nm; λ_{em} = 620 nm.

DFT Calculation Results

Non-Protonated Pyrylazo (output structure)

Charge = 1 Multiplicity = 1

C	3.86134	-1.15831	-0.35782
C	4.91245	-0.28993	-0.18791
O	4.66439	0.99447	0.14273
C	3.41768	1.47658	0.31739
C	2.33696	0.63876	0.16212
C	2.53657	-0.71192	-0.17944
C	3.38938	2.92045	0.67267
C	6.36235	-0.59076	-0.33113
C	1.38213	-1.66465	-0.33135
N	0.10953	-0.96515	-0.50587
N	-0.72688	-1.33592	0.35392
C	-2.03584	-0.81002	0.22443
C	-2.93732	-1.18522	1.23372
C	-4.24518	-0.72887	1.20739
C	-4.67322	0.10557	0.1595
C	-3.77526	0.47782	-0.86082
C	-2.46401	0.0189	-0.82512
O	-5.96951	0.49423	0.21618
C	-6.49774	1.32395	-0.81899
H	4.06967	-2.18725	-0.62991
H	1.33535	1.02788	0.29175
H	2.36129	3.26352	0.79269
H	3.9357	3.09333	1.60644
H	3.87646	3.51355	-0.10901
H	6.51212	-1.63925	-0.59122
H	6.80557	0.03761	-1.1113
H	6.88903	-0.37604	0.60495
H	1.56572	-2.28695	-1.21845
H	1.35843	-2.33206	0.54688
H	-2.59525	-1.83807	2.03104
H	-4.95555	-1.00835	1.97757
H	-4.0932	1.10927	-1.68146
H	-1.76612	0.28323	-1.61199
H	-7.542	1.49168	-0.55704
H	-5.97459	2.28612	-0.86298
H	-6.44204	0.82551	-1.79341

Protonated Pyrylazo (output structure)

Charge = 2 Multiplicity = 1

C	-3.84993	-1.21278	0.1971
C	-5.00468	-0.4785	0.01228
O	-4.90555	0.85199	-0.17151
C	-3.74014	1.52007	-0.18583
C	-2.55927	0.82088	-0.00548
C	-2.60277	-0.56579	0.18899
C	-3.8942	2.98017	-0.40083
C	-6.39995	-0.98231	-0.01157
C	-1.35739	-1.39941	0.41262
N	-0.1342	-0.61354	0.36822
N	0.89645	-1.26237	0.0002
C	2.19298	-0.79948	-0.02678
C	3.18881	-1.67404	-0.54538
C	4.49467	-1.26283	-0.59518
C	4.84953	0.03393	-0.12425
C	3.84688	0.90466	0.40167
C	2.53667	0.49119	0.44984
O	6.12573	0.33496	-0.20853
C	6.65072	1.61449	0.2344
H	-3.93531	-2.28362	0.3471
H	-1.62043	1.35874	-0.01348
H	-2.92298	3.47521	-0.41664
H	-4.41242	3.17119	-1.3477
H	-4.50734	3.4164	0.39661
H	-6.42615	-2.06138	0.14308
H	-6.99244	-0.49265	0.77005
H	-6.87163	-0.74523	-0.97236
H	-1.43723	-1.84538	1.41864
H	-1.35005	-2.23695	-0.30451
H	2.92101	-2.66508	-0.90424
H	5.27857	-1.90291	-0.98411
H	4.11336	1.88622	0.77329
H	1.7699	1.138	0.86016
H	7.7179	1.56029	0.03164
H	6.19743	2.42442	-0.34222
H	6.47599	1.73882	1.30587
H	0.79299	-2.24178	-0.30954

Non-Protonated Pyrylazo (orbitals) **Protonated Pyrylazo (orbitals)**

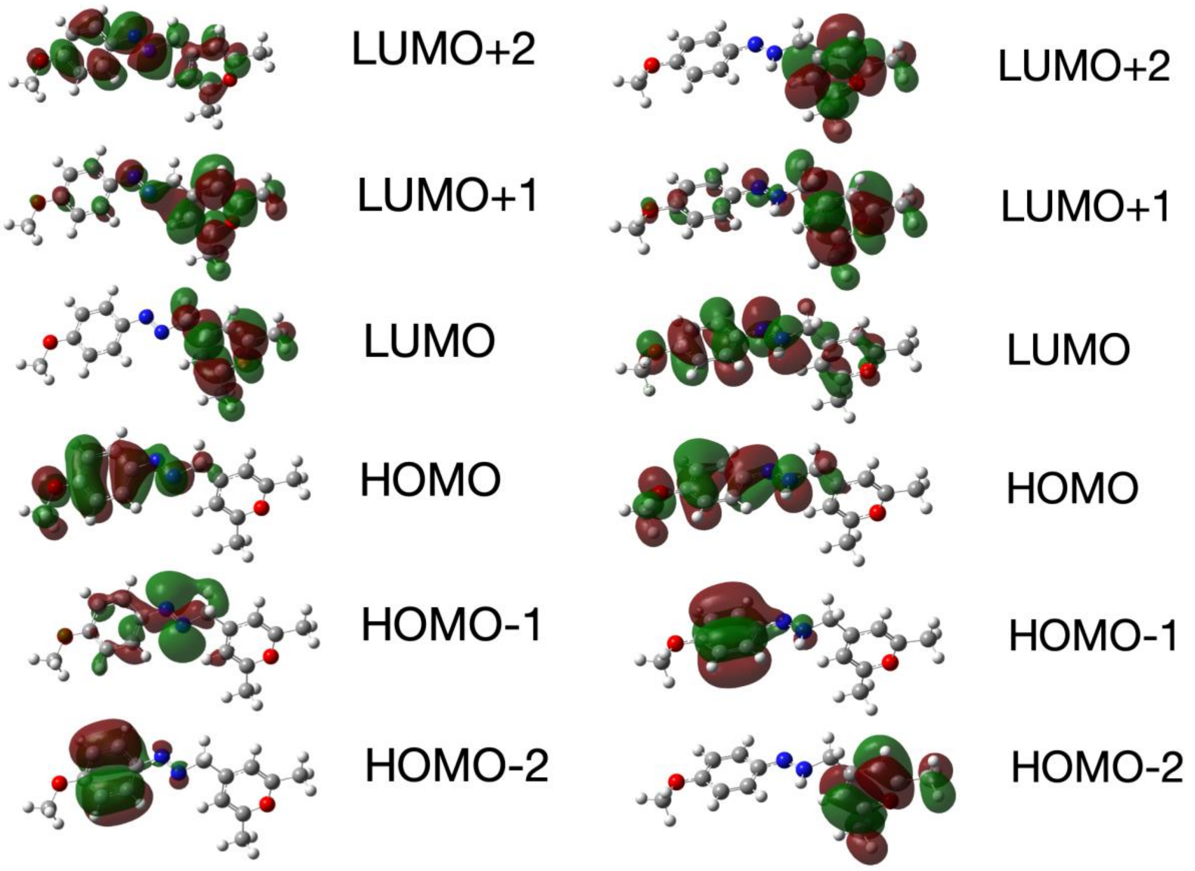

Ortho modified Pyrylazo (output structure)

Charge = 1 Multiplicity = 1

C	-2.56778	-0.49112	-0.18207
C	-2.52811	0.87411	-0.02777
C	-3.71898	1.58859	0.20696
C	-4.92084	0.8592	0.26917
C	-4.91428	-0.50912	0.10317
H	-1.56526	1.36536	-0.10182
H	-5.86804	1.35757	0.44434
O	-3.74692	-1.13949	-0.11852
C	-1.41205	-1.40815	-0.43019
H	-1.59515	-1.93803	-1.37738
H	-1.36273	-2.16845	0.36472
C	-6.09601	-1.41626	0.13211
H	-6.66637	-1.26777	1.05457
H	-6.76431	-1.19368	-0.70706
H	-5.78385	-2.45924	0.06769
C	-3.69937	3.07124	0.40981
H	-3.47047	3.2887	1.46185
H	-2.92198	3.54538	-0.19444
H	-4.66557	3.5256	0.18063
C	2.0539	-0.66224	-0.06868
C	2.36379	0.49779	-0.81611
C	3.06877	-1.31519	0.64608
C	3.65403	0.985	-0.82589
H	1.58593	0.99016	-1.38852
C	4.37268	-0.82718	0.64615
H	2.81646	-2.21155	1.20387
C	4.67227	0.32967	-0.09326
H	3.92173	1.86834	-1.39624
H	5.13795	-1.34578	1.2087
N	0.78456	-1.25235	0.01428
N	-0.16765	-0.64775	-0.54233
C	6.98762	0.3126	0.54119
H	7.21325	-0.68991	0.16182
H	7.83521	0.9722	0.36049
H	6.77399	0.2705	1.61484
O	5.8928	0.89671	-0.1722

Pyrylazo-isomer (output structure)

Charge = 1 Multiplicity = 1

C	-3.84993	-1.21278	0.1971
C	-5.00468	-0.4785	0.01228
O	-4.90555	0.85199	-0.17151
C	-3.74014	1.52007	-0.18583
C	-2.55927	0.82088	-0.00548
C	-2.60277	-0.56579	0.18899
C	-3.8942	2.98017	-0.40083
C	-6.39995	-0.98231	-0.01157
C	-1.35739	-1.39941	0.41262
N	-0.1342	-0.61354	0.36822
N	0.89645	-1.26237	0.0002
C	2.19298	-0.79948	-0.02678
C	3.18881	-1.67404	-0.54538
C	4.49467	-1.26283	-0.59518
C	4.84953	0.03393	-0.12425
C	3.84688	0.90466	0.40167
C	2.53667	0.49119	0.44984
O	6.12573	0.33496	-0.20853
C	6.65072	1.61449	0.2344
H	-3.93531	-2.28362	0.3471
H	-1.62043	1.35874	-0.01348
H	-2.92298	3.47521	-0.41664
H	-4.41242	3.17119	-1.3477
H	-4.50734	3.4164	0.39661
H	-6.42615	-2.06138	0.14308
H	-6.99244	-0.49265	0.77005
H	-6.87163	-0.74523	-0.97236
H	-1.43723	-1.84538	1.41864
H	2.92101	-2.66508	-0.90424
H	5.27857	-1.90291	-0.98411
H	4.11336	1.88622	0.77329
H	1.7699	1.138	0.86017
H	7.7179	1.56029	0.03164
H	6.19743	2.42442	-0.34222
H	6.47599	1.73882	1.30587
H	0.79299	-2.24178	-0.30954

Ortho modified Pyrylazo

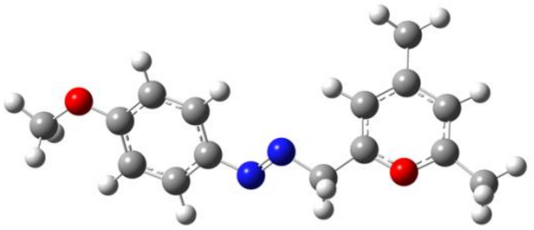

Pyrylazo-isomer

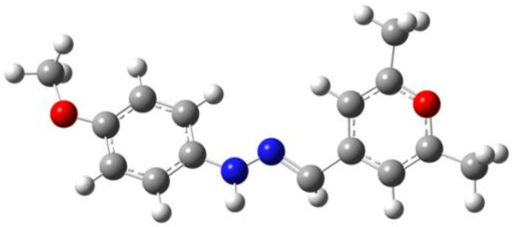

Cell assays

The experiments to identify the compound concentration and reaction condition for the Pyrylazo fluorescent probe were performed to achieve the highest fluorescence without triggering cell death. The study using higher probe concentrations led to cell alterations, with round morphology instead of the characteristic elongated fibroblast shape and cell death.

Table S1. Bright-field and epifluorescence microscopy images of mouse fibroblast cells (5×10^4 cells/well) treated for 2 h with 5000-1000 µM of Pyrylazo.

Concentration	Bright-field	Green channel
5000 µM	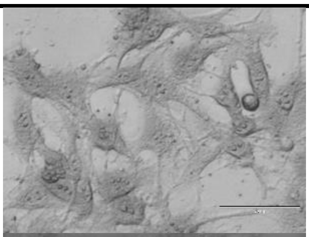	NF
3500 µM	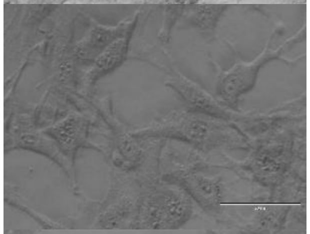	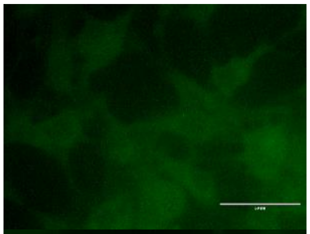
2000 µM	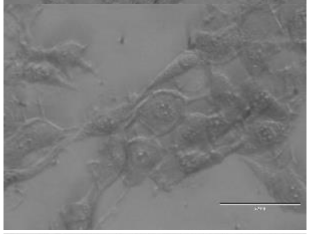	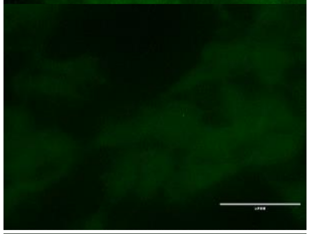
1000 µM	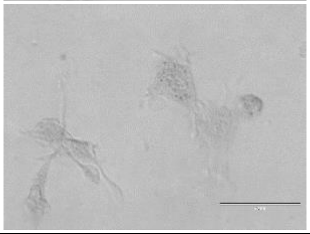	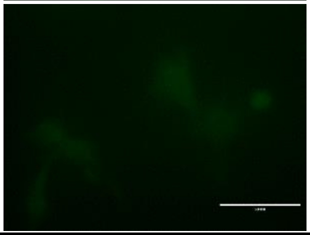

NF: no fluorescence. Bars represent 50 µm.

Table S2. Bright-field and epifluorescence microscopy images of mouse fibroblast cells (5×10^4 cells/well) treated for 2 h with 0.02% (v/v) Tween-20 and 5000-100.10 µM of Pyrylazo.

Concentration	Bright-field	Green channel
5000 µM	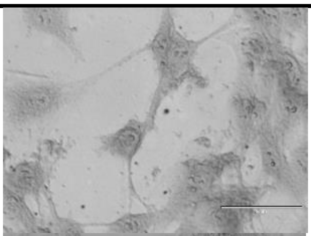	NF
3500 µM	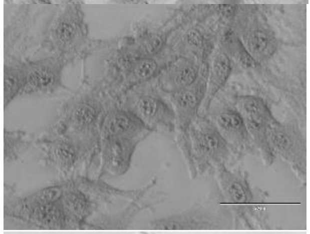	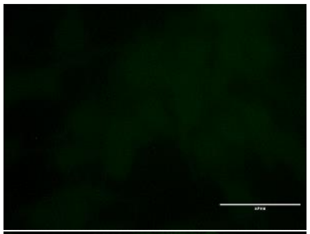
2000 µM	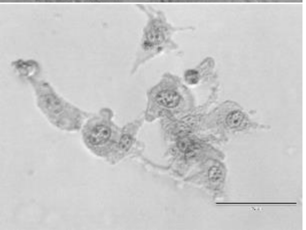	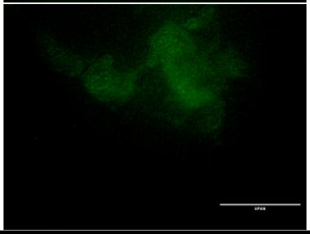

NF: no fluorescence. Bars represent 50 µm.

Table S3. Bright-field and epifluorescence microscopy images of mouse fibroblast cells (5×10^4 cells/well) treated for 2 h with 100-0.10 µM of Pyrylazo.

**Concentration**	**Bright-field**	**Green channel**	**Red channel**
100 µM	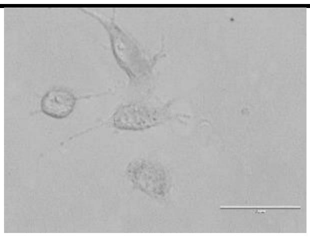	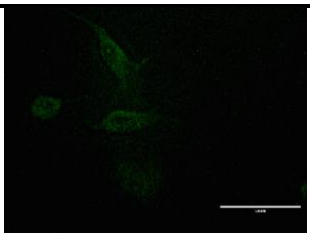	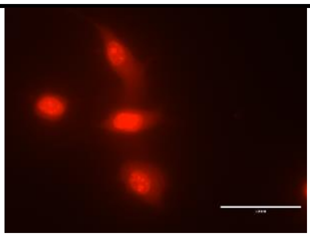
10 µM	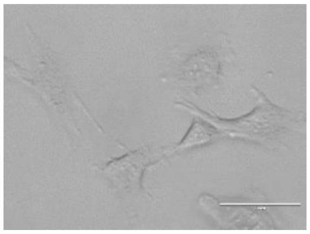	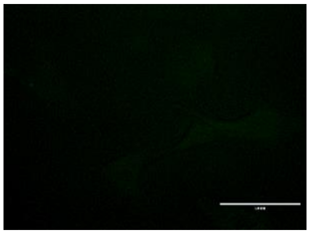	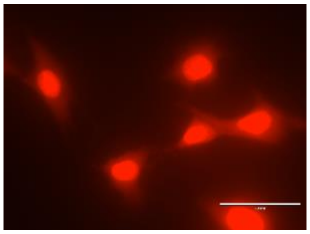
1.0 µM	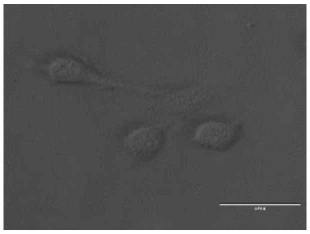	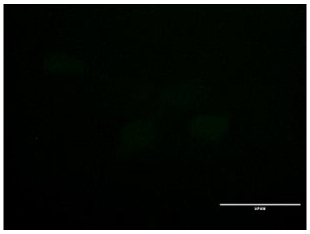	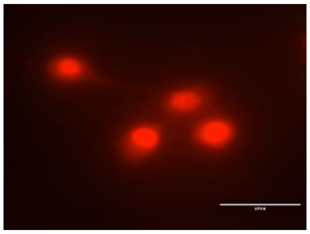
0.10 µM	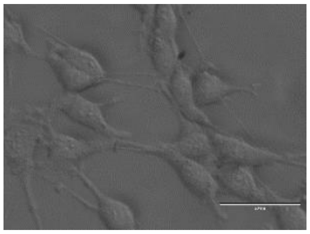	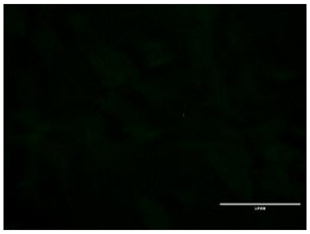	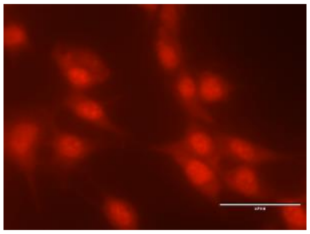
Negative control	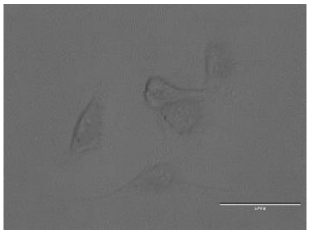	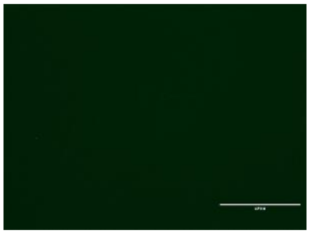	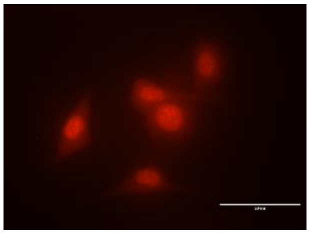

Bars represent 50 µm.

Crystallographic data results

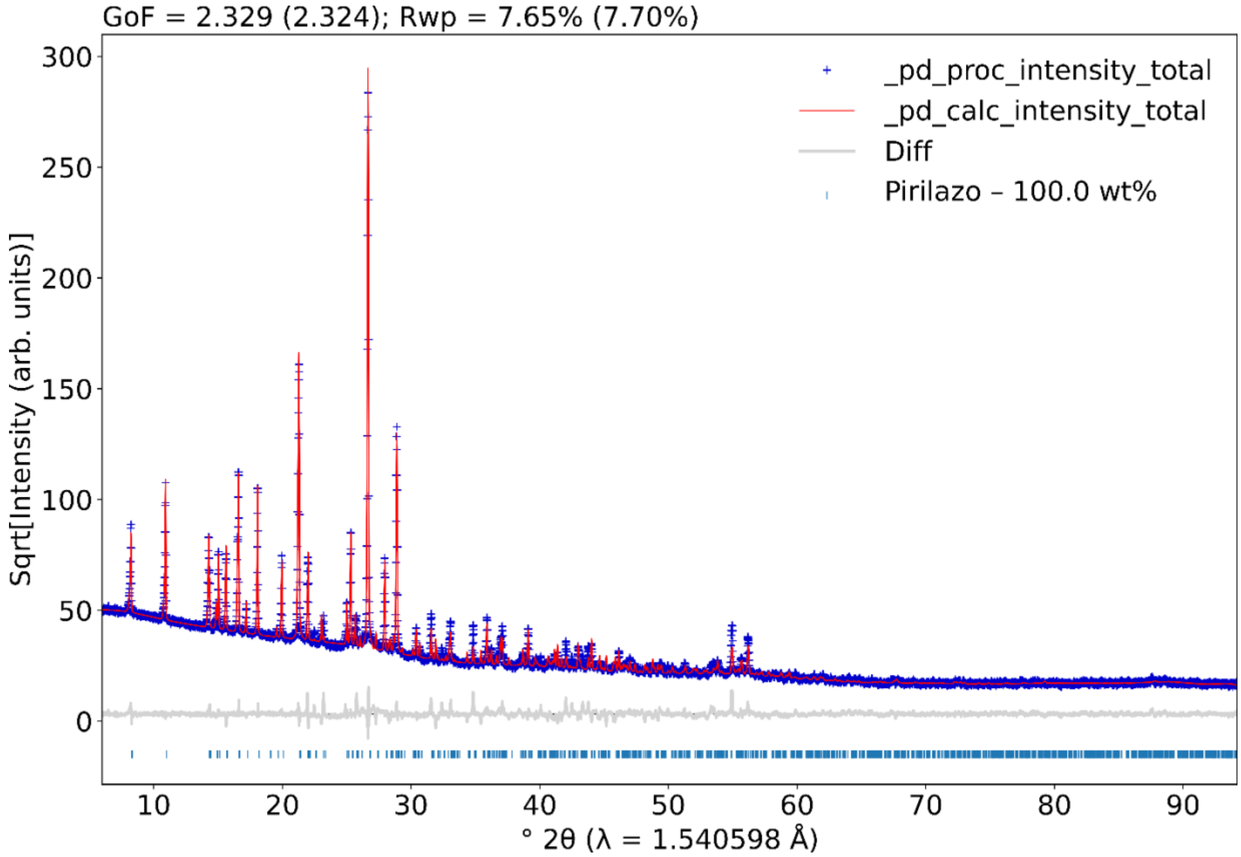

Figure SC1. Rietveld plot of Pyrylazo generated using pdCIFplotter.[1] The blue crosses indicate the experimental intensities, the red line is the calculated diffractogram based on the determined crystal structure, and the gray line is the difference between the observed and calculated diffractograms. The vertical bars at the bottom indicate Bragg reflections.

Table SC1. Crystal data and details of the crystal structure determination.

Chemical formula	$C_{15}H_{17}N_2O_2$, BF_4
Formula weight (g mol^{-1})	344.12
Crystal system	Otrhorhombic
Space group	$Pna2_1$ (Nr. 33)
a, *b*, *c* (Å)	21.303(3), 12.3763(16), 6.6701(9)
Volume (Å^3)	1758.6(4)
Z, Z´	4, 1
ρ_{calc} (g cm^{-3})	1.2997(3)
T (K)	298
Data collection	
Diffractometer	STADI P
Monochromator	Ge(111)
Wavelength (Å)	1.54056
2θ range (°)	6.000-94.185
Step size (°)	1.05
Time per step (s)	30
Refinement	
Number of data points	5880
Number of contributing reflections	899
R_p (%)	3.285
R_{wp} (%)	7.650
χ^2	2.329

Table SC2. Atomic Coordinates for Pyrylazo

Atom	x	y	z
B1	0.3150(3)	0.2627(6)	0.7303(11)
F2	0.2894(4)	0.1796(8)	0.8279(16)
F3	0.3342(4)	0.2321(12)	0.5368(14)
F4	0.2667(4)	0.3377(9)	0.7052(18)
F5	0.3617(4)	0.2985(10)	0.8566(16)
C6	0.5004(4)	-0.1152(7)	0.6982(9)
C7	0.4312(3)	-0.1509(5)	0.7103(5)
N8	0.5130(4)	-0.0068(7)	0.654(2)
C9	0.3830(3)	-0.0776(7)	0.7159(5)
C10	0.4170(4)	-0.2598(6)	0.7152(6)
N11	0.5681(3)	0.0170(7)	0.6940(11)

C12	0.3231(3)	-0.1129(5)	0.7258(7)
C13	0.3572(3)	-0.2920(5)	0.7247(7)
C14	0.5769(3)	0.1289(5)	0.7057(7)
O15	0.3110(3)	-0.2197(6)	0.7299(7)
C16	0.2642(5)	-0.0485(10)	0.699(3)
C17	0.3343(6)	-0.4038(8)	0.7306(11)
C18	0.5286(4)	0.2018(6)	0.7017(7)
C19	0.6376(4)	0.1641(7)	0.7270(7)
C20	0.5413(3)	0.3109(5)	0.7155(8)
C21	0.6497(3)	0.2762(5)	0.7390(8)
C22	0.6021(3)	0.3485(5)	0.7355(8)
O23	0.6201(3)	0.4529(5)	0.7517(8)
C24	0.5713(16)	0.5325(9)	0.7394(9)
H1	0.5162(4)	-0.1271(7)	0.8312(9)
H2	0.5296(4)	-0.1543(7)	0.6162(9)
H3	0.3954(3)	-0.0030(7)	0.7131(5)
H4	0.4504(4)	-0.3118(6)	0.7124(6)
H7	0.3683(6)	-0.4529(8)	0.7057(11)
H8	0.3188(6)	-0.4070(8)	0.5956(11)
H9	0.2993(6)	-0.4209(8)	0.8155(11)
H10	0.4842(4)	0.1918(6)	0.6868(7)
H11	0.6752(4)	0.1214(7)	0.7284(7)
H12	0.5104(3)	0.3673(5)	0.7121(8)
H13	0.6896(3)	0.3115(5)	0.7532(8)
H14	0.5881(16)	0.6040(9)	0.7546(9)
H15	0.5417(16)	0.5224(9)	0.8464(9)
H16	0.5488(16)	0.5286(9)	0.6150(9)
H17	0.2796(5)	0.0232(10)	0.7230(3)
H18	0.2381(5)	-0.0665(10)	0.8114(3)
H19	0.2515(5)	-0.0574(10)	0.5619(3)

Table SC3. Bond Length Data

Atom 1	Atom 2	Bond Length (Å)
B1	F2	1.33(1)
B1	F3	1.41(1)
B1	F4	1.40(1)
B1	F5	1.38(1)
C6	C7	1.54(1)
C6	N8	1.40(1)
C9	C7	1.37(1)
C10	C7	1.38(1)
N11	N8	1.24(1)
C9	C12	1.350(9)
C13	C10	1.34(1)
C14	N11	1.40(1)
O15	C12	1.35(1)
C16	C12	1.50(1)
O15	C13	1.331(9)
C17	C13	1.47(1)
C18	C14	1.37(1)
C19	C14	1.37(1)
C20	C18	1.38(1)
C21	C19	1.41(1)
C20	C22	1.383(9)
C21	C22	1.353(9)
O23	C22	1.352(9)
O23	C24	1.43(3)

Table SC4. Valence Angle Data

Atom 1	Atom 2	Atom 3	Bond Angle (°)
F3	B1	F2	111.1(8)
F4	B1	F2	105.7(8)
F5	B1	F2	104.2(8)
F4	B1	F3	106.5(8)
F5	B1	F3	116.0(8)
F5	B1	F4	113.1(8)
C7	C6	N8	118.0(7)
C6	C7	C9	121.9(5)
C6	C7	C10	119.4(5)
C9	C7	C10	118.7(5)
C6	N8	N11	111.4(9)
C7	C9	C12	119.7(6)
C13	C10	C7	120.0(6)
C14	N11	N8	112.0(8)
O15	C12	C9	120.0(6)
C16	C12	C9	127.9(7)
O15	C12	C16	111.4(7)
O15	C13	C10	120.4(6)
C17	C13	C10	126.8(6)
O15	C13	C17	112.8(6)
C18	C14	N11	123.4(6)
C19	C14	N11	116.5(6)
C19	C14	C18	120.1(6)
C13	O15	C12	121.2(6)
C20	C18	C14	119.8(6)
C21	C19	C14	119.3(6)
C18	C20	C22	121.3(6)
C19	C21	C22	120.8(6)
C21	C22	C20	118.8(6)
O23	C22	C20	126.5(6)
O23	C22	C21	114.7(6)
C24	O23	C22	116.5(8)

References

(1) Rowles, M. R. *J. Appl. Crystallogr*. **2022**, *55* (3), 631.
